# Oxygen availability and body mass modulate ectotherm responses to ocean warming

**DOI:** 10.1038/s41467-023-39438-w

**Published:** 2023-06-27

**Authors:** Murray I. Duncan, Fiorenza Micheli, Thomas H. Boag, J. Andres Marquez, Hailey Deres, Curtis A. Deutsch, Erik A. Sperling

**Affiliations:** 1 Earth and Planetary Science, Stanford University, Stanford, CA USA; 2 Oceans Department, Hopkins Marine Station, Stanford University, Pacific Grove, CA USA; 3 Department of Environment, University of Seychelles, Anse Royale, Seychelles; 4 Blue Economy Research Institute, University of Seychelles, Anse Royale, Seychelles; 5 Department of Ichthyology and Fisheries Science, Rhodes University, Makhanda, South Africa; 6 Stanford Center for Ocean Solutions, Stanford University, Pacific Grove, CA USA; 7 Department of Earth and Planetary Sciences, Yale University, New Haven, CT 06511 USA; 8 Earth Systems, Stanford University, Stanford, CA USA; 9 Department of Geosciences and the High Meadows Environmental Institute, Princeton, NJ USA

**Keywords:** Ecophysiology, Climate-change ecology, Marine biology

## Abstract

In an ocean that is rapidly warming and losing oxygen, accurate forecasting of species’ responses must consider how this environmental change affects fundamental aspects of their physiology. Here, we develop an absolute metabolic index (Φ_A_) that quantifies how ocean temperature, dissolved oxygen and organismal mass interact to constrain the total oxygen budget an organism can use to fuel sustainable levels of aerobic metabolism. We calibrate species-specific parameters of Φ_A_ with physiological measurements for red abalone (*Haliotis rufescens*) and purple urchin (*Strongylocentrotus purpuratus*). Φ_A_ models highlight that the temperature where oxygen supply is greatest shifts cooler when water loses oxygen or organisms grow larger, providing a mechanistic explanation for observed thermal preference patterns. Viable habitat forecasts are disproportionally deleterious for red abalone, revealing how species-specific physiologies modulate the intensity of a common climate signal, captured in the newly developed Φ_A_ framework.

## Abstract (web summary)

This study presents an absolute metabolic index that quantifies how ocean temperature, dissolved oxygen and organismal mass interact to constrain the oxygen budget an organism can use to fuel aerobic metabolism. The index is calibrated with physiological measurements from purple sea urchin and red abalone and the authors test if the index can delimit the distribution of these two species.

## Introduction

The ocean’s physical and chemical environment is currently in a period of accelerated change. Ocean average temperatures^1^ and the magnitude and frequency of extreme temperature events^2^ are increasing while at the same time the ocean is losing oxygen^3^, threatening the persistence of biodiversity^4–6^. Ectothermic marine organisms may respond to this environmental change by shifting their distributions^7^, maximum size and growth rates^8^, and/or phenology^9^, which ultimately influence population persistence and productivity and the livelihoods of ocean resource users^10^. Proactive management and adaptation approaches aimed at mitigating impacts of climate-induced resource declines are therefore critically needed^11,12^, but are hampered by our inability to forecast the direction and magnitude of species-specific responses^13,14^. Because organismal responses to ocean warming and deoxygenation are mediated through their physiology, frameworks that quantify effects of this environmental change on vital physiological processes can serve as a gauge for how future environmental conditions will be processed, and thereby improve forecasting accuracy on focal species^15–17^.

An organism’s aerobic metabolism is a vital physiological process that generates a major component of the energy required to perform fitness related activities like growth, reproduction, and competition via the production of ATP, which consumes oxygen in the process^18,19^. The amount of energy that can be allocated to these activities is therefore fundamentally constrained by an organism’s capacity to supply (*S*) oxygen to its aerobic metabolic pathways above that level required to sustain its basal oxygen demand (*D*)^20,21^. Two of the most pervasive extrinsic drivers on an ectothermic organism’s oxygen demand (*D*) and capacity for oxygen supply (*S*) are ocean temperature and dissolved oxygen bioavailability, respectively^22,23^. Species have therefore evolved such that their physiological machinery is designed to supply sufficient oxygen to fuel sustainable levels of aerobic metabolism, given the prevailing environmental conditions they are exposed to^24^. As the ocean continues to warm and lose oxygen, species responses must be partly determined by their capacity to maintain a sustainable balance between oxygen supply (*S*) and demand (*D*), which is captured by a metabolic index (Φ)^21,25^ (Eq. 1).

**Fig. 1:**
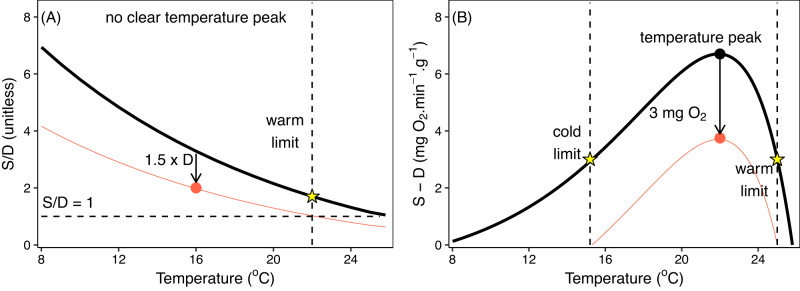
Shape of thermal relationships for different expressions of an organism’s oxygen balance in saturated water. **A** When the balance between oxygen supply (S) and basal demand (D) is expressed as a ratio (Solid black line), no clear temperature peak is identified if supply is a single monotonic function of temperature and only a single thermal limit can be identified (star). Note that the relationship between supply and temperature may not always be monotonic with temperature^27, 29, 30^. Thermal optima (lowest *PO*_*2crit*_) arise from the multi-step nature of O_2_ supply, which can be readily reproduced by the factorial form of the metabolic index^30^, but both the absolute and factorial forms shown here assume a single temperature-dependent supply function. **B** The same data expressed as the difference between S and D (solid black line) often yields a thermal peak and both warm and cold thermal limits can be estimated (stars). When additional energetic processes are performed the available oxygen balance decreases (red lines) in A and B. If these energetic processes are vital for performance a species’ thermal limits are defined as those temperatures where oxygen supply falls to match elevated oxygen demand (equal to a value of one in A and zero in B).The oxygen requirement of these additional processes can be quantified and removed from the total oxygen budget in B but must be measured in relation to demand in A, which is challenging if thermal sensitivities differ (i.e. additional energetic process does not equal 1.5 x basal demand across whole temperature range).

The metabolic index (Φ) is the ratio between oxygen supply and demand $\left(\frac{S}{D}\right)$. This index accounts for effects of environmental oxygen availability, temperature, and body mass and represents the greatest possible proportional increase in oxygen supply above basal requirements (Fig. 1A). For an environment to be ecologically sustainable Φ must be greater than a minimum critical level (Φ_crit_) representing the oxygen level required to fuel sustainable levels of aerobic metabolism^25^. Because the parameters of Φ are species-specific, calibrated with experimental physiological measurements, it is a general framework that can account for varied species responses to ocean warming and deoxygenation. Indeed, Φ has proved useful for explaining the spatial variability in extinction rates across the Permian Triassic boundary^26^, contemporary species biogeography patterns^25,27^ and the interannual productivity variation of marine fish^28^ to date.

While Φ is useful for delimiting either warm or cool distribution edges, it can fail to delimit a species’ entire thermal envelope (both warm and cool edges) because of its factorial form, when oxygen supply is governed by a single monotonic exponential function (e.g., Fig. 1A, Fig. 2 in ref. ^25^). In such cases Φ will always increase towards either warm or cool sides of a species distribution provided oxygen remains saturated, as is the case in surface waters (Fig. 1A). In some cases however, respirometry data indicate that Φ can discern a thermal optima^27,29,30^ which can arise from the multi-step nature of oxygen supply^30^, but the generality across species remains unknown. This hinders predictions of how climate-mediated distribution shifts may manifest across a heterogeneous environment, such as upwelling-driven marine ecosystems, which are characterised by localised warming and cooling patterns. Furthermore, not all energetic processes that use oxygen have the same thermal sensitivity as basal oxygen demand, and organisms rather allocate a specific amount of oxygen to additional processes from a total oxygen budget^31,32^. Expressing an organisms’ oxygen budget in absolute terms $\left(S-D\right)$ rather than factorial $\left(\frac{S}{D}\right)$ permits quantifying the energy budget into consumable units and can produce thermal optima^33^ (e.g., Fig. 1B) even in cases where a monotonic exponential rise in oxygen supply with temperature exists^30^. An absolute form of Φ could thus prove a powerful new tool to further enhance our ability to predict future ecological impacts due to climate change signals in the ocean.

**Fig. 2:**
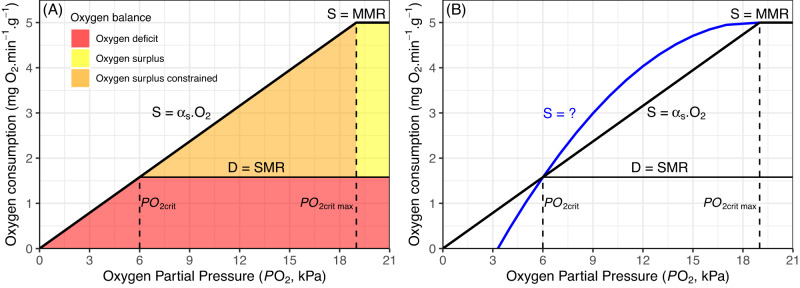
The influence of oxygen availability (*PO*_*2*_, kPa) on oxygen supply (*S*) at a single temperature. **A** When oxygen availability is high, supply (S) is not constrained, and potential to consume excess oxygen is maximized (yellow area). As environmental oxygen decreases below *PO*_*2crit* max_, oxygen supply is limited at a rate equal to the oxygen supply capacity (α_*S*_) and potential for excess oxygen consumption is constrained (orange area). When oxygen supply (*S*) equals demand (*D*) the critical oxygen partial pressure (*PO*_*2crit*_) is reached, below which there is a deficit of oxygen required to fuel basal demand (red area). **B** Shape of oxygen limitation relationships when α_*S*_ is constant (linear thick black line) or variable (non-linear blue line) yet *PO*_*2crit*_ and *PO*_*2crit max*_ are identical.

Here we build on the foundations of Φ and recent experimental evidence of the environmental oxygen and thermal dependence of aquatic species’ oxygen supply and demand to develop an absolute metabolic index (Φ_A_). To test the explanatory power of Φ_A_ we calibrate species-specific parameters with physiological measurements following acute thermal exposure for the purple sea urchin (*Strongylocentrotus purpuratus*) and red abalone (*Haliotis rufescens*), two sedentary benthic marine invertebrates that experience dynamic environmental temperature and oxygen variability throughout the California Current Large Marine Ecosystem (CCLME). *Haliotis rufescens* is a culturally important and once economically valuable fishery species whose numbers have recently collapsed, while at the same time numbers of *S. purpuratus* have exploded and decimated kelp forests in the region^34^. We compare Φ_A_ predictions for optimal temperatures (*T*_*opt*_ Φ_A_), considering oxygen availability and organismal mass, with experimental and aquaculture evidence of both species’ optimal temperatures (*T*_*opt*_). We further test if Φ_A_ can delimit the core biogeographic distribution of these two species. Finally, we use Φ_A_ to make predictions of how future climate change scenarios in the CCLME may drive distributional changes of both species, which can ultimately inform climate adaptation for fisheries management and marine conservation.

## Results

### Deriving an absolute metabolic index (Φ_A_)

The effect of environmental oxygen availability in constraining an organism’s maximum rate of oxygen consumption is depicted by a line of oxygen limitation (thick solid lines in Fig. 2A, B). At a given temperature, this maximum capacity to supply (*S*) oxygen to an organism’s tissue is the product of the oxygen available in the environment (*PO*_*2*_) and its oxygen supply capacity (α_*S*_ – a rate constant for gas exchange between water and organism), up towards a ceiling (*PO*_*2crit max*_) where additional oxygen provides no increases and the maximum metabolic rate (MMR) is reached (Fig. 2A, B)^24,35^. The minimum level of oxygen demand (*D*) required to sustain basic functioning is quantified as an organism’s basal or standard metabolic rate (SMR) (thin solid line in Fig. 2A, B) and remains constant between normoxia and the line of oxygen limitation. When oxygen demand (*D*) equals maximum oxygen supply (*S*) the critical oxygen partial pressure (*PO*_*2crit*_) is reached, below which there is insufficient oxygen obtainable to fuel SMR and conditions become unsuitable for long-term survival (Fig. 2A, red area). The slope of the line of oxygen limitation (α_*S*_) is sensitive to temperature because the biophysical mechanisms governing it (ventilation, circulation, diffusion) are themselves dependent on temperature as well as organismal body mass (because of its pervasive effect on organismal demand for oxygen)^19,25,35^.

The effect of temperature, environmental oxygen and body mass on the ratio of oxygen supply (S) to oxygen demand (D) is quantified by a metabolic index (Φ) following Eq. 1^25^, where demand is an organism’s standard metabolic rate (α_*D*_) per unit body mass (*B*) and its scaling exponent (*δ*) at a given the temperature (*T*), while supply is the product between the amount of oxygen available in the environment (*PO*_*2*_) and oxygen supply capacity (α_*S*_) given the temperature (*T*) per unit body mass (*B*) and its scaling exponent (*σ*).

1 $$\Phi=\frac{S}{D}=\frac{{\alpha }_{{S}_{(T)}}.P{O}_{2}.{B}^{\sigma }}{{\alpha }_{{D}_{(T)}}.{B}^{\delta }\,}$$

An absolute version of the metabolic index (Φ_A_) is therefore oxygen supply minus oxygen demand following Eq. 2. Φ_A_ will equal zero when the amount of oxygen in the environment (*PO*_*2*_) is equivalent to an organism’s *PO*_2crit_ and a functional relationship for oxygen supply capacity (α_*S*_) at any temperature is derived as $\big(\frac{{{\alpha }_{D}}_{(T)}}{P{O}_{{2{crit}}_{(T)}}}\big)$. Both demand (α_*D*_) and *PO*_*2crit*_ are experimentally measured permitting supply capacity (α_*S*_) to be quantified. The mass scaling exponent for supply (σ) is therefore captured as the difference between the mass scaling coefficients for demand (δ) and for *PO*_2crit_ (ε).

2 $${\Phi }_{A}=S-D=(\Phi -1).\,D=P{O}_{2}.\,{B}^{\delta -\varepsilon }\left(\frac{{\alpha }_{{D}_{(T)}}}{P{O}_{2cri{t}_{(T)}}}\right)-{\alpha }_{{D}_{(T)}}.\,{B}^{\delta }$$

The major assumptions of Φ_A_ are that *PO*_*2crit max*_ is close to normoxia (21 kPa for coastal species) and is not temperature sensitive, which is supported by recent experimental evidence^35^, and that oxygen supply (*S*) declines linearly (constant α_*S*_) with decreasing oxygen availability between *PO*_*2crit*_ and *PO*_*2crit max*_ (e.g., Fig. 2B, thick solid black line). Evidence for the second assumption is more limited, with recent studies indicating some fish species exhibit curvilinear decreases in oxygen supply with progressive hypoxia^36^ and that this shape can change with hypoxia acclimation^37^ (e.g., Fig. 2B, solid blue line) as organisms make morphological and physiological adjustments to maintain oxygen supply^38^. In such cases direct comparisons of Φ_A_ values for a species at different temperatures and *PO*_*2*_ levels are inaccurate. Because the thermal dependance of α_*S*_ still holds^36^, we facilitate comparison across the oxygen – temperature space by normalizing Φ_A_ per oxygen level (Φ_A_’) to between [0,1] following Eq. 3 in methods. Thus, final values of Φ_A_’ range from zero (Φ_A_ <= zero) to one - the temperature where Φ_A_ is greatest for each level of oxygen availability organisms are exposed to in the ocean.

### Respirometry experiments

**Fig. 3:**
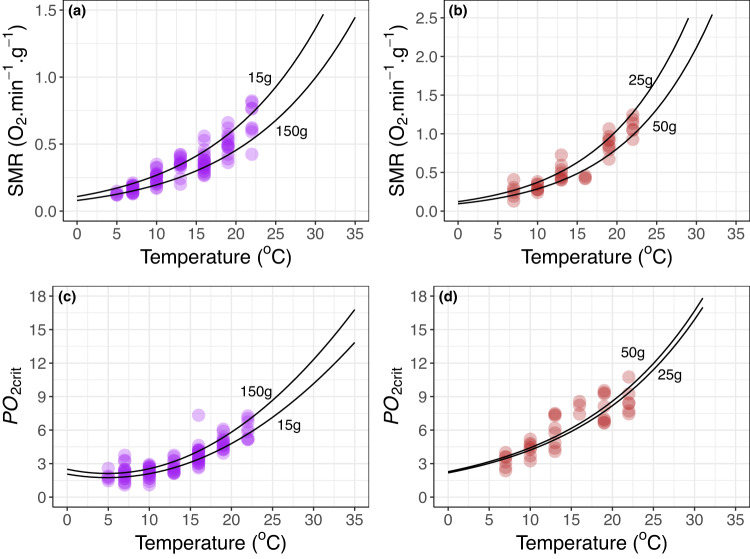
Physiological measurements. Oxygen demand taken as standard metabolic rate (SMR; **a**, **b**) and critical oxygen partial pressure (*PO*_*2crit*_; **c**, **d**) data for the purple sea urchin, *S. purpuratus* (purple points) and red abalone, *H. rufescens* (red points) across experimental temperatures. Solid lines represent best fit Arrhenius models for SMR (**a**, **b**) and *PO*_*2crit*_ fit with a quadratic (**c**) or exponential (**d**) model for *S. purpuratus* and *H. rufescens*, respectively. Models are illustrated for two different size classes corresponding to the smallest and largest wet weight (g) of experimental specimens. Source data are provided as a Source Data file.

Oxygen demand (standard metabolic rate, SMR) and critical oxygen partial pressure (*PO*_*2crit*_) increased with temperature for both *S. purpuratus* and *H. rufescens* (Fig. 3a–d). Temperature effects on SMR were well approximated by Arrhenius models with mass-specific allometric exponents of −0.14 and −0.38 estimated for *S. purpuratus* and *H. rufescens*, respectively (*SI Appendix*, Fig. S1 and S2). *PO*_*2crit*_ was slightly higher than what would be predicted at colder temperatures (< = 7 °C) by Arrhenius models for both species, especially for *S. purpuratus* where temperature had little influence on *PO*_*2crit*_ between 5 and 10 °C. The temperature effect on *PO*_*2crit*_ was well approximated by a quadratic polynomial for *S. purpuratus* and a general exponential model for *H. rufescens*. Wet mass had little effect on *PO*_*2crit*_ for both species, with weak allometric mass exponents estimated as 0.08 and 0.07 for *S. purpuratus* and *H. rufescens*, respectively (*SI Appendix*, Fig. S1 and S2). These modeled relationships were used to predict Φ_A_ for each species and test its utility.

### Φ_A_ predictive utility

**Fig. 4:**
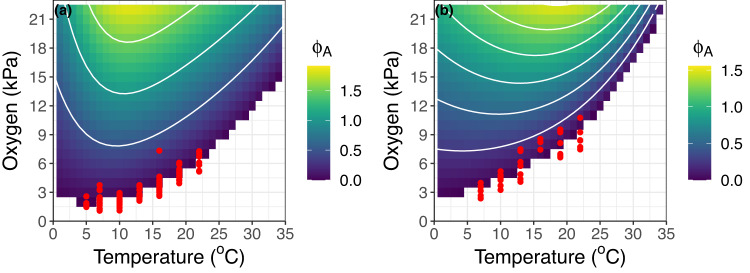
Φ_A_ predictions across temperature-oxygen space. Φ_A_ model predictions (viridis color scale) for *S. purpuratus* (**a**) and *H. rufescens* (**b**) across a temperature and oxygen state-space. Φ_A_ values correspond to the median size (wet mass) of experimental organisms (*S. purpuratus* = 67.5 g, *H. rufescens* = 37.5 g), white lines are Φ_A_ contours, and red points correspond to experimental *PO*_*2crit*_ measurements where Φ_A_ = zero. Source data are provided as a Source Data file.

Φ_A_ predicts clearly defined thermal optima (*T*_*opt*_ Φ_A_) for both *S. purpuratus* and *H. rufescens* (Fig. 4a, b). Φ_A_ decreases with progressive deoxygenation via its constraint on maximum rate of oxygen supply until basal levels of oxygen demand cannot be maintained and *PO*_*2crit*_ is reached (Φ_A_ = zero). Predicted temperature dependent *PO*_*2crit*_ corresponds to experimentally quantified *PO*_*2crit*_ (red points in Fig. 4).

### Optimal Temperature

**Fig. 5:**
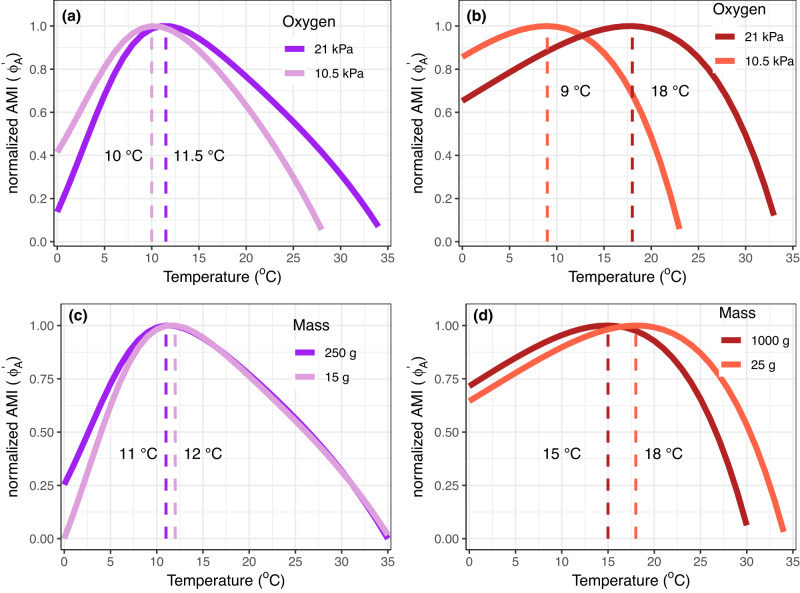
Variable optimum temperatures. Effect of oxygen availability (**a**, **b**) and mass (**c**, **d**) on the optimal temperature predictions from normalized absolute metabolic index (Φ_A_’) models for *S. purpuratus* (purple) and *H. rufescens* (red). Source data are provided as a Source Data file.

Optimal temperature predictions for Φ_A_ (*T*_*opt*_ Φ_A_) are consistent with optimal temperature estimates from published experimental studies for both species. Φ_A_ models predict that in oxygen-saturated seawater (21 kPa) and for a median sized specimen used in this study (*S. purpuratus* = 67.5 g wet mass, *H. rufescens* = 37.5 g wet mass), the optimum temperature (*T*_*opt*_ Φ_A_) is 11.5 °C for *S. purpuratus* and 18 °C for *H. rufescens* (Fig. 5a, b). These *T*_*opt*_ Φ_A_ predictions are remarkably consistent with *T*_*opt*_ results from aquaculture and experimental biology studies for both species. For *S. purpuratus* weighing around 80 g, the recommended rearing temperature to optimize gonadal production in aquaculture systems is 12 °C^39^ and although working on larvae, the same authors also recommend rearing temperatures of between 11 and 14 °C for optimal growth and survival^40^. A *T*_*opt*_ Φ_A_ for *H. rufescens* of 18 °C corresponds well with studies on smaller sized specimens in fully aerated experimental systems. Optimal growth rates for juvenile *H. rufescens* are reported at 18 °C for specimens from southern California^41^ and around 17.3 °C for specimens from Morro Bay^42^ while the preferred temperature of warm acclimated juveniles from Baja, Mexico is estimated at 18.8 °C^43^. Working with gametes of *H. rufescens*, Boch et al.^44^ found that warmer sea water (18 °C) was beneficial as it mitigated the negative effects of low pH on fertilization success. Some studies have however found deleterious effects of warmer temperatures on *H. rufescens*, manifested via increased susceptibility to abalone withering syndrome^45–47^. However, studies comparing growth and survival both with and without exposure to the agent of the withering syndrome suggests this is not a result of direct thermal stress or physiological impairment but rather increased disease infection efficacy in warmer water^48^.

### Oxygen and mass effects on optimum temperature

Optimal temperatures predicted by Φ_A_ models vary with both oxygen and organismal mass, consistent with results of empirical studies. Specifically, *T*_*opt*_ Φ_A_ decreases as oxygen availability decreases (Fig. 5a, b) or organismal mass increases (Fig. 5c, d), and this effect is more pronounced for *H. rufescens* than *S. purpuratus*. This is in close agreement with the results of Steinarsson et al.^42^ who found that the optimal growth temperature for *H. rufescens* decreases with size from 17.3 °C for specimens with 33 mm shell lengths to 14.5 °C for those with a 98 mm shell length. This size and oxygen dependance of *T*_*opt*_ Φ_A_ may explain why studies on larger *H. rufescens* report decreased performance metrics in warmer waters around 18 °C. Larger *H. rufescens* specimens (~90 mm shell length, compared to the average of 55 mm in this study) held in a flow-through sea-water system with natural oxygen variability showed better growth, survival and performance at ~13.5 °C (2.5 °C below the average temperature at La Jolla, California) compared to temperatures ~18.5 °C (2.5 °C above)^45^, while substantially larger specimens (~180 mm shell length) showed compromised fecundity and the onset of withering syndrome when exposed to 18 °C water^46^. *H. rufescens* is predicted to be particularly sensitive to low oxygen availability, with a 50% drop in oxygen corresponding to a 9 °C cooler shift in *T*_*opt*_ Φ_A_ and complete collapse in Φ_A_ at temperatures just above 20 °C (Fig. 5b). In contrast, *T*_*opt*_ Φ_A_ for *S. purpuratus* only decreases 1.5 °C for the same 50% drop in oxygen (Fig. 5a), and *T*_*opt*_ Φ_A_ only decreases 1 °C between our large and small size classes (Fig. 5c, note this is not directly comparable to the difference in size classes for *H. rufescens*).

### Metabolic niche

**Fig. 6:**
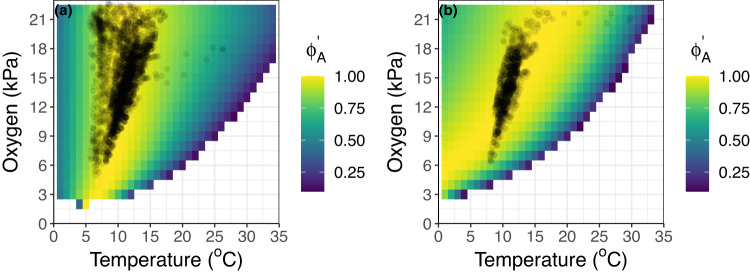
Φ_A_’ explains metabolic niche. OBIS geo-referenced occurrences (black points) for *S. purpuratus* (**a**) and *H. rufescens* (**b**) matched with corresponding temperature and oxygen conditions from the ROMS ocean model and layered over Φ_A_’ predictions across the temperature-oxygen state-space (viridis colors). Source data are provided as a Source Data file.

Normalized Φ_A_ (Φ_A_’) predictions across a temperature and oxygen space approximate the conditions where *S. purpuratus* and *H. rufescens* naturally occur (Fig. 6a, b). For both species, most wild occurrence locations are in areas where the associated monthly temperature and oxygen availability result in a Φ_A_’ close to 1.0 (Fig. 6, yellow area) indicating that these organisms track temperatures that optimize capacity for excess oxygen supply given the prevailing environmental oxygen levels. This coupling suggests that Φ_A_’ has predictive utility at demarcating viable habitat considering the prevailing temperature and oxygen conditions. In our analyses, the agreement between species occurrences and Φ_A_’ is tighter for *S. purpuratus* compared to *H. rufescens*, which may result in projecting suitable habitat for *H. rufescens’* in areas where they do not naturally occur. As discussed below, such misfit can result from the accuracy of the primary model inputs, namely 1) experimental respirometry data, 2) modeled or measured seafloor oxygen and temperature conditions, and 3) biogeographic occurrence data.

### Current biogeography

**Fig. 7:**
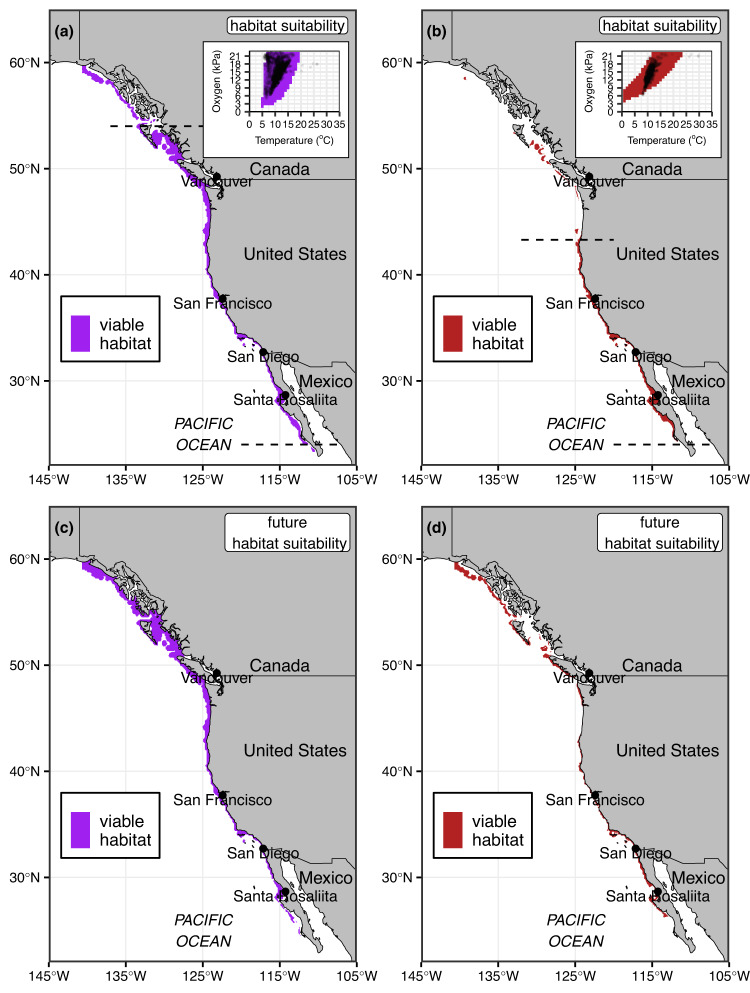
Spatial modelling. Spatial predictions of contemporary (1995–2010, **a**, **b**) and future (2071–2100 under the RCP 8.5 emissions scenario, **c**, **d**) viable habitat for *S. purpuratus* (purple) and *H. rufescens* (red) derived by classifying the minimum normalized Φ_A_ value for each cell as suitable above a 0.849 threshold for *S. purpuratus* and 0.928 threshold for *H. rufescens* (inserts). Dashed lines delimit core continuous distribution predictions. Note that the future distribution of *H. rufescens* is hard to visualize at this large spatial scale because it loses habitat at depth and hugs the coastline closely.

Normalized Φ_A_ (Φ_A_’) delimits the core contemporary spatial distribution of both species. We estimate an occurrence threshold (Φ_A_’ value above which 99% of geo-referenced occurrence points fall) of 0.849 for *S. purpuratus* and 0.928 for *H. rufescens* (Fig. 7, inserts and *SI Appendix*, Fig. S3). That is, both species naturally occur only in regions where the prevailing temperature is close to the temperature where Φ_A_ is maximized given environmental oxygen levels. Projecting these thresholds across the minimum monthly Φ_A_’ value for each grid cell of the ocean model spatially delimits a core continuous stretch of habitat that coincides with the documented biogeographic range of both species accurately (Fig. 7a, b). Overall, these spatial projections provide further evidence that Φ_A_’ has predictive power for demarcating viable habitat and therefore might be used to forecast climate change effects. However, the certainty of predictions needs to be considered within the context of tightness of fit between Φ_A_’ habitat suitability and occurrences. The index overestimates viable habitat for *H. rufescens* (Fig. 6 and Fig. 7 insert) and hence may overestimate the species’ contemporary distribution and future climate mediated distribution contractions.

Spatial models predict a continuous contemporary range for *S. purpuratus* from central/southern Baja, Mexico in the south to the Alaska, USA and British Columbia, Canada border in the north and large patches further north, in accordance with its documented core distribution^49^. *S. purpuratus* has been reported slightly further north than the model domain^50^ and its reported southernmost occurrence point near Bahia Tortugas, Mexico^51^ is slightly further north than the modelled southern limit. The core continuous distribution prediction for *H. rufescens* between the Oregon/California border in the USA to southern Baja is also in accordance with its documented distribution^52^. While the species is less abundant in central Baja, it has been reported in literature at the southern tip of the peninsula^52^. Spatial models for *H. rufescens* also predict small, isolated patches of viable habitat (which can be an artefact of such modelling approaches) around Vancouver Island, which remarkably match seven documented occurrences of the species, believed to have been transported via drift kelp^53^. Isolated patches of predicted viable habitat for *H. rufescens* further north are likely unviable and a result of spatial over-projection due to poorer fit with occurrences.

### Future biogeography changes

Ocean warming and deoxygenation is predicted to disproportionally reduce the viable habitat for *H. rufescens* compared to *S. purpuratus* in their core distribution range. Forecasts up to 2100 predict that the viable habitat distribution of *S. purpuratus* will shift north but will remain relatively unaffected throughout its core range (Fig. 7c). In contrast, the distribution of *H. rufescens* is predicted to become more constrained and fractured, particularly along California (Fig. 7d). Deoxygenation in deeper water and warming temperatures in shallower waters are predicted to contract *H. rufescens’* depth distribution by 2100. Although the viable habitat is predicted to contract and fracture, the model also predicts the growth of additional habitat in northern Washington, Vancouver Island, Haida Gwaii, and further north but the potential for persistence in these areas is unknown.

## Discussion

### Φ_A_ discerns the entire metabolic niche of species

We find that the absolute metabolic index (Φ_A_) accurately depicts how environmental temperature, oxygen availability, and organismal mass interact to constrain the viable habitat for two common marine invertebrates, *S. purpuratus* and *H. rufescens*, across the California Current Large Marine Ecosystem (CCLME). A key advantage of Φ_A_ demonstrated here is its ability to discern a thermal optimum, and how this changes with body mass and deoxygenation, permitting the delimitation of a species’ entire biogeographic envelope – which is not typically achieved with the metabolic index (Φ) in its factorial form^21,54^. Congruency between Φ_A_ predictions of thermal optima and experimental studies further corroborates this framework and supports the theory that the balance between oxygen supply and demand has an important role in regulating thermal responses, even if oxygen limitation may not be the proximate cause or set critical thermal limits^55–57^. Rather, the Φ_A_ framework highlights that the temperature where excess oxygen supply is maximized varies depending on organismal mass and environmental oxygen level. Thus, aquatic organisms can exhibit ecological thermal responses even in the absence of environmental temperature change (i.e., if oxygen levels change) or in a direction not explained by temperature alone as they seek conditions that maximize oxygen provision.

Φ_A_ predicts that, for the species investigated here, the optimal temperature for excess oxygen supply is cooler for larger sized individuals. Within-species negative correlations between body size and temperature is a common ecological observation termed James’s rule but the mechanism driving this pattern is not certain^58^. Physiological mechanisms are posited to underly this pattern because growth rates account for a significant component of total energy budgets^59^ and the temperature where growth rate is greatest decreases with body size^60,61^. This pattern is driven in Φ_A_ predictions by the smaller mass-scaling exponent for oxygen supply (σ = *δ − ε*) than demand (*δ*) causing Φ_A_ to decrease per unit body mass as organisms get bigger. The effect of this decrease is amplified at warmer temperatures because the thermal sensitivity of demand is greater than supply, such that even if *PO*_*2crit*_ scales close to zero with mass^62^ the peak Φ_A_ will exhibit a cool temperature shift. While we cannot pinpoint the specific mechanism it is notable that this pattern is consistent with observations of temperature preference with size in aquatic ectotherms^63^.

Φ_A_ predicts deoxygenation will drive organisms to cooler temperatures, providing a mechanistic explanation for observed thermal responses to hypoxia. Indeed, experimental evidence for cooler temperature preference in less oxygenated water is reported for the Atlantic cod (*Gadus morhua*)^64^, and the Carmine shiner (*Notropis percobromus*)^65^. This pattern is driven, in Φ_A_ models, because oxygen supply (*S*) is the product of oxygen supply capacity (α_*S*_), which increases with temperature, and environmental oxygen availability (*PO*_*2*_), resulting in a smaller relative increase in oxygen supply with temperature when environmental oxygen is low. Furthermore, deoxygenation itself is directly detrimental to organismal functioning with small decreases making habitat inhospitable and triggering distribution shifts^66,67^. Organisms in ocean regions that are warming and losing oxygen – the climate change signal in the CCLME and other upwelling ecosystems – are thus particularly vulnerable as not only will deoxygenation shift the *T*_*opt*_ Φ_A_ cooler, but ocean warming will concurrently move temperatures further away from *T*_*opt*_ Φ_A_. If temperature and oxygen change are heterogenous across space (e.g., with depth and latitude), such as in the CCLME, viable habitat can become fractured, limiting potential latitudinal range shifts^27,28^. The synergistic negative effects of concurrent deoxygenation and warming highlighted by the Φ_A_ framework provides further evidence that temperature and oxygen availability must be considered in tandem when assessing climate resilience of marine organisms.

We find close matches between *T*_*opt*_ Φ_A_ in oxygen-saturated water and temperatures where physiological processes such as growth are maximized for both species. While we do not explicitly measure maximum metabolic rate (MMR) the Φ_A_ in oxygen saturated sea water should theoretically provide a similar value to absolute aerobic scope (MMR - SMR) if modelled oxygen supply (*S*) is similar to MMR, for which some evidence exists^34^. Numerous studies have however found mismatches between the temperatures where absolute aerobic scope is maximized and preferred temperatures^68^ or temperatures where growth efficiency is maximized^69^ for fishes. The Φ_A_ framework shows that the temperature where excess oxygen supply is greatest decreases with deoxygenation (Fig. 5a, b), and this concept is important to consider when evaluating why absolute aerobic scope in temperature-only models may have limited explanatory power. This is particularly relevant when respirometry experiments are performed in oxygen-saturated water, but the study organism naturally occurs in low oxygen environments, such as mangroves for Barramundi *Lates calcarifer*^68,70^ or deep water for Atlantic halibut *Hippoglossus hippoglossus*^69,71^. Although more research is required on the generalizability of Φ_A_, the potential for environmental oxygen availability to alter the thermal optima for maximum oxygen provision highlights that experiments where maximal rates of respiration are measured should consider the comparability between experimental oxygen levels and those the study organism is naturally exposed to.

While the Φ_A_ framework presented here for *S. purpuratus* and *H. rufescens* elucidates thermal optima, provides a mechanism behind the oxygen and mass dependence of thermal optima, and delimits both species’ contemporary biogeography, it is not without important caveats. Only two invertebrate species were tested and whether the Φ_A_ framework generalizes to other taxa is unknown. Even among just these two taxa, metabolic niche predictions appear more accurate for *S. purpuratus* compared to *H. rufescens*. There are multiple possible reasons for this difference. This poorer fit for *H. rufescens* may be because we did not measure physiological traits at the coldest temperature (5 °C) for *H. rufescens* and hence modelled a more monotonic thermal relationship for *PO*_*2crit*_ data, resulting in broader Φ_A_’ curves per oxygen level and potentially erroneous suitable Φ_A_’ predictions at cold temperatures (<7 °C). We also found geographic bias for this species in the OBIS occurrence data we used, with few documented occurrences of *H. rufescens* recorded in the warmer Mexican ocean despite the species occurring there^52^. In fact, *H. rufescens* had less than half the geo-referenced occurrence points in OBIS as *S. purpuratus*, and both species showed geographic areas where the species currently exists but is simply not recorded in OBIS. Like all modeling approaches, Φ_A_’ is sensitive to data inputs, and the predictive power for a given species will continue to improve as the three main data inputs—biogeographic occurrences, species-specific physiological parameters, and measured or modeled oceanographic conditions—continue to be better documented. A final plausible explanation is that the contemporary distribution of *H. rufescens* is more constrained by additional factors other than temperature and oxygen availability, such as resource competition, fishing pressure, predation, or life history and habitat specialization^72,73^. Forecasted future distribution changes based solely on environmental variables therefore need to be considered in the context of over predicting contemporary biogeographic distribution and predicted future range contractions.

### Moving from models to management

Climate-driven warming and deoxygenation in the CCLME is predicted to reduce and fracture *H. rufescens*’ distribution through constraints on Φ_A._ This is in addition to a multitude of other interacting stressors – e.g., overfishing, kelp deforestation and associated loss of food and habitat, and disease - that have already contributed to collapsing the once productive *H. rufescens* fishery^38,74^. In contrast, climate-driven reduction in Φ_A_ is predicted to have relatively little effect on *S. purpuratus*, whose numbers have recently exploded along the California margin, shifting kelp forests to barren states^38^. Caution must be exercised when considering such forecasts as they do not include higher spatial or temporal levels of environmental variability or mechanisms that confer resilience such as generational adaptation or acclimation following chronic exposure, where rates return to baseline levels after the initial increase during acute exposure (which are only captured in this study)^75^. Nonetheless, despite the nature of future predictions being inherently imprecise they can serve as a comparative tool to identify what species and areas are relatively vulnerable to warming and deoxygenation and focus improving management regulations. Currently implemented management approaches to maintain or rebuild wild abalone stocks and kelp forest ecosystems include the implementation of marine reserves to re-establish top-down, predatory control of urchin populations, urchin culling^76^, fishing moratoria, and kelp restoration^74^, but the efficacy and scalability of these approaches is debated^77^. Ensuring the persistence of abalone populations, as well as other ecologically, economically, and culturally important species and whole kelp forest ecosystems, will require prioritizing these management approaches in areas predicted to provide relative refugia under future climate scenarios, such as, for abalone, northern Baja (Fig. 7d). In parts of the CCLME around central California, however, population and ecosystem restoration attempts may be compromised by ocean deoxygenation and warming over which local management has no direct control.

Our key findings here are in accordance with a recent first-principles integration of environmental oxygen availability into a framework showing mass-and temperature-effects on levels of metabolism^78^. That is, lower environmental oxygen reduces the temperature sensitivity of higher metabolic rates relative to lower ones, shifting the temperature with the greatest oxygen balance cooler, and that higher metabolic rates in larger bodied organisms will be disproportionally constrained at higher temperatures. Although rates of aerobic metabolism above basal levels are not specifically incorporated, the Φ_A_ framework does quantify the total oxygen budget available to fuel such rates. Importantly Φ_A_ is calibrated with species-specific experimental physiological measurements and can make species-specific predictions of how oxygen budgets will be constrained across the entire range of temperature and environmental oxygen values organisms are exposed to in the wild. Moving forward, the generalizable Φ_A_ framework will be key tool to forecast climate responses of focal species as the ocean continues to warm and lose oxygen.

## Methods

### Absolute metabolic index - Φ_A_

Parameters from *PO*_*2crit*_ and SMR (α_D_) models are used to predict species specific values of Φ_A_ given the prevailing temperature (*T*), environmental oxygen (*PO*_*2*_) and organismal mass (B) following Eq. 2 where *δ* is the allometric mass scaler for SMR and *ε* is the allometric mass scaler for *PO*_*2crit*_ (equation presented in the introduction).

To compare Φ_A_ values at various temperature (T) and oxygen levels (O) accounting for plasticity in the shape of the limiting oxygen level line, we normalize Φ_A_ by rescaling between zero and one for each oxygen level (whole kPa unit) following Eq. 3. The resulting Φ_A_’ thus ranges from zero (lowest Φ_A_) to one (highest Φ_A_) for each kPa level (22–0 kPa) where Φ_A_ has a positive value, permitting an easier comparison of *T*_*opt*_ Φ_A_ across the oxygen scape and accounting for potential non-linear limiting oxygen level curves, assuming the thermal dependance of α still holds^35^.

3 $${{\Phi }_{A}}^{{\prime} }=\frac{{\Phi }_{{A}_{(O,T)}}-\,min({\Phi }_{{A}_{(O)}})}{max({\Phi }_{{A}_{(O)}})-min({\Phi }_{{A}_{(O)}})}$$

Species-specific experimental physiological measurements for *PO*_*2crit*_ and SMR across a range of temperatures and body masses are therefore first required.

#### Experimental Protocol

Ethical approval for experiments was not required as the study organisms are classified as invertebrates and were not obtained from the wild. All other research procedures comply with relevant ethical regulations. Purple sea urchin *Strongylocentrotus purpuratus* (*n* = 97, wet mass range 14.8–151.3 g) and red abalone *Haliotis rufescens* (*n* = 38, wet mass range 26.9 – 51.3 g) were obtained from Monterey Abalone Company prior to each experimental trial, where they had been held in underwater cages exposed to natural ocean temperatures and fed a natural kelp diet. Respirometry techniques were used to quantify standard metabolic rate (SMR) and critical oxygen partial pressure (*PO*_*2crit*_) for each specimen at a specific test temperature. A single trial involved placing freshly obtained specimens directly into respirometry chambers (see *SI Appendix*, table S1 for detailed methods following in ref. ^79^) at 12.7 °C (close to the average seawater temperature in Monterey Bay, at the depths where these organisms are commonly found in ref. ^80^) and subsequently adjusting the water temperature at a rate of ~1 °C per hour until a test temperature of 5, 7, 10, 13, 16, 19 or 22 °C was reached and held constant (+/−0.4 °C). Specimens were given ~24 h to adjust to novel respirometry chamber and temperature conditions and purge any digesting food, after which intermittent flow respirometry (510 min measure – 10 min flush) was run for a further ~24 h to quantify SMR. Oxygen levels in chambers were continuously recorded with a FireStingO2 fiber optic oxygen meter (FSO2-4, Pyro Science GmbH) connected to oxygen sensor spots (Pyroscience OXSP5) positioned in a circulation loop with bare optical fibers (SPFIB-BARE, Pyro Science GmbH) and logged with Pyro oxygen logger software. After ~24 h of intermittent flow respirometry the *PO*_*2crit*_ phase of the trial began by switching off the flush pump so that oxygen could be depleted within respirometer chambers via the specimen’s aerobic metabolism until no oxygen remained. Each specimen was used once to obtain SMR and *PO*_*2crit*_ measurements at a single temperature. The trial was then terminated, test specimens frozen and later thawed to weigh to the nearest hundredth of gram (wet mass). We also dried test specimens at 50 °C for ~24 h, measured their dry mass, subsequently ashed them at 450 °C for three hours in a muffle furnace, and measured their ashed mass to calculate a metabolic mass (dry minus ashed mass) which is recorded in the metadata for completeness. A blank was run in an empty respirometry chamber in parallel for each trial.

#### Quantifying SMR

The mass specific oxygen consumption rate (*M*O_2_, mg O_2_. min^−1^. g^−1^) of each measurement period was calculated using Eq. 4^81^ where V_re_ is the total volume of the respirometer in ml; M is the mass of the specimen in g expressed as ml; W is mass of the specimen in g, $\frac{\Delta [{{{\mbox{O}}}}_{2{{{\rm{a}}}}}]}{\Delta t}$ is the oxygen consumption rate during the measurement period and $\frac{\Delta [{{{\mbox{O}}}}_{2{{{\rm{b}}}}}]}{\Delta t}$ is the corresponding oxygen consumption rate of the of an empty chamber (background respiration).

4 $$M{{{{{{\rm{O}}}}}}}_{2}=\left(\left(\frac{{{{{{{\rm{V}}}}}}}_{{{{{{\rm{re}}}}}}}-\,{{{{{\rm{M}}}}}}}{{{{{{\rm{W}}}}}}}\right)\left(\frac{\varDelta [{{{{{{\rm{O}}}}}}}_{{{{{{\rm{2a}}}}}}}]}{\varDelta {{{{{\rm{t}}}}}}}\times 60\right)\right)-\left(\left(\frac{{{{{{{\rm{V}}}}}}}_{{{{{{\rm{re}}}}}}}-\,{{{{{\rm{M}}}}}}}{{{{{{\rm{W}}}}}}}\right)\left(\frac{\varDelta [{{{{{{\rm{O}}}}}}}_{{{{{{\rm{2b}}}}}}}]}{\varDelta {{{{{\rm{t}}}}}}}\times 60\right)\left(\frac{{{{{{{\rm{V}}}}}}}_{{{{{{\rm{re}}}}}}}}{({{{{{{\rm{V}}}}}}}_{{{{{{\rm{re}}}}}}}-{{{{{\rm{M}}}}}})}\right)\right)$$

Oxygen consumption rates of each measurement period were calculated using the calc_rate function in the respR package^82^. Erroneous oxygen consumption rate measurements were filtered out using an R^2^ > 0.95 quality threshold (0.9 for three small urchins at 7 °C) and removing measurements associated with background respiration rates outside the 95^th^ and 5^th^ quantiles of all measurements, which may have occurred during temperature adjustments in the respirometry system. SMR was estimated as the 0.1 quantile of all remaining oxygen consumption measurements^22^.

#### Quantifying PO_2crit_

The oxygen drawdown curve from the *PO*_*2crit*_ phase of the experiment was split into five-minute segments with mass-specific oxygen consumption rates calculated for each segment as per SMR protocol. All *M*O_2_ measurements (SMR and *PO*_*2crit*_ phases of the trial) were paired with corresponding average oxygen concentration values converted to percent saturation using the conv_o2 function from the respirometry package^83^. We define *PO*_*2crit*_ as the the lowest level of oxygen (percent saturation) at which an animal can maintain SMR^84^ and estimated it with the calcO2crit function^22^. The maximum number of points (max.nb.MO2.for.reg) to fit the regression below *PO*_*2crit*_ was taken as the number of points that had the best fit (R^2^) and ensured the intercept was negative. We corroborated the robustness of our *PO*_*2crit*_ approach by comparing results with a method where *PO*_*2crit*_ is taken as the oxygen partial pressure where oxygen supply capacity (α_*S*_) is maximized, following Seibel et al.^24^ (*SI Appendix*, Fig. S4).

#### Calibrating Φ_A_

##### SMR

The effect of mass and temperature on a species’ oxygen demand (α_D_) quantified as standard metabolic rate (SMR) is described by Eq. 5^19^ where $a$ is the rate coefficient, *B* is mass in grams and *δ* is its allometric scaling exponent, *E* is activation energy, *k* is Boltzmann’s constant (eV/K) and *T* is absolute temperature in Kelvin^85^.

5 $${SMR}={a.\,B}^{\delta }{.\,e}^{E/{kT}}$$

We first divided SMR data with its Arrhenius function$\left({e}^{\frac{E}{k.T}}\right)$ to remove the effect of temperature and fit a power function through the relationship with organismal mass to estimate *δ*^21^. The Arrhenius function was estimated from the slope of the linear relationship between the natural logarithm of SMR data and inverse temperature (*k.T*). We took the constants of the SMR model; $a$ and *E* as the back transformed intercept and slope respectively from the linear relationship between the natural logarithm of mass-corrected SMR $\left(\frac{{SMR}}{{B}^{\delta }}\right)$ and the inverse of *k.T* (product of Boltzmann constant and temperature in Kelvin).

##### PO_2crit_

We followed the same approach as SMR for modeling *PO*_*2crit*_ data for each species but did not fit Arrhenius models across the whole temperature range as the relationship can break down at colder temperatures where *PO*_*2crit*_ may be higher than predicted^27,29^, possibly due to reduced oxygen diffusivity in colder water^86^. We first converted *PO*_*2crit*_ data from percent saturation to oxygen partial pressure (kPa) using the conv_o2 function from the respirometry package^83^ including the experiment temperature, salinity = 35 PSU, and standard atmospheric pressure (1013.2) as arguments. For both species, *PO*_*2crit*_ data fit Arrhenius relationships at temperatures above 7 °C. We therefore estimate the allometric mass scaling exponent *ε* from the power relationship between temperature standardized *PO*_*2crit*_ (*PO*_*2crit*_ divided by the Arrhenius function for temperatures above 7 °C) and organismal mass (*B*). We then fit more flexible models through the relationship between mass corrected *PO*_*2crit*_ $\left(\frac{{{PO}}_{2{crit}}}{{B}^{\varepsilon }}\right)$ and temperature (°C). *PO*_*2crit*_ data for *S. purpuratus* was well approximated by a quadratic polynomial and *H. rufescens* by a general exponential model.

#### Spatial modelling

##### Ocean models

To map the spatial and temporal distribution of species-specific Φ_A_ values in the CCLME we used high-resolution (12 km) oceanic simulations made with the Regional Oceanic Modeling System (ROMS)^87^. The ocean model domain extends from 15° N to 60° N, 4000 km offshore from the coast of America and has 21 depth bins spaced ten meters apart^88^. Model simulations are conducted for a hindcast period from 1995 to 2010 as well as a future climate projection for the end of this century (2071–2100) under the RCP 8.5 emissions scenario. The future climate projection applies surface and open boundary condition anomalies averaged across multiple models in the CMIP5 archive, following procedures outlined by Howard et al.^88^. Mean monthly temperature (°C), oxygen (*u*M) and salinity (PSU) are interpolated from the native s-coordinate to regular 10-meter intervals for analysis. Gridded bathymetry data were obtained from the General Bathymetric Chart of the Oceans (GEBCO) website at a 15 arc second (450 m) resolution. For each month we extracted the layer of environmental variables closest to the ocean floor by overlapping grid cells of the ocean model with bathymetry cells greater than −150 metres (~maximum depth of occurrence for both species) and extracting values from the depth bin of the ocean model that most closely matched the depth of bathymetry layer. We calculated the water pressure at depth in mbar using the swPressure function in the oce package^89^ and added atmospheric pressure (1013.25 mbar). We then calculated the oxygen bioavailability in partial pressure units (kPa) using the conv_o2 function including corresponding temperature, salinity, and pressure as arguments.

##### Occurrence data

We obtained geo-referenced occurrence data for *S. purpuratus* and *H. rufescens* from the Ocean Biodiversity Information System (OBIS) using the robis robis,^90^. Overall, we retrieved 4595 *S. purpuratus* and 1582 *H. rufescens* occurrences. We matched these occurrences to the same grid structure as the ocean model and removed duplicates resulting in 230 unique geo-referenced occurrence points for *S. purpuratus* and 91 for *H. rufescens*.

##### Spatial modelling

We paired each geo-referenced occurrence point with corresponding monthly temperature and oxygen availability from the ocean models and estimated the corresponding Φ_A_ value. We identified habitat suitability thresholds for Φ_A_’ as the value where 99% of all occurrence points fell above. To map each species distribution, we produced a gridded layer of the minimum monthly Φ_A_’ value for each species and applied their corresponding habitat suitability thresholds (i.e. Φ_A_’ values above thresholds that were considered suitable). We followed the same process for the future ocean model forecasts to predict how each species’ distribution may change.

All analyses were carried out in R version 4.0.3^91^ with the following packages; ggspatial^92^, raster^93^, marmap^94^, rnaturalearth^95^, sf^96^, viridis^97^ and tidyverse^98^ in addition to those cited in text.

### Reporting summary

Further information on research design is available in the Nature Portfolio Reporting Summary linked to this article.

## Supplementary information

Supplementary information

Peer Review File

Reporting Summary

## Data Availability

The raw respirometry data generated during experiments and the processed data used for the analyses in this study have been deposited in the Zenodo database here: 10.5281/zenodo.7899786^99^. Source data are provided with this paper.

## Source data

Source data

## Code availability

The R script used to analyze the data in this study has been deposited in the Zenodo database here: 10.5281/zenodo.7899786^99^.
