# Supplementary material for: Oxygen availability and body mass modulate ectotherm responses to ocean warming
Source: Nat Commun. 2023 Jun 27;14:3811. doi: 10.1038/s41467-023-39438-w (PMC10300008; doi:10.1038/s41467-023-39438-w)
Supplement: Supplementary file 1 — Supplementary information [file 41467_2023_39438_MOESM1_ESM.pdf]

Supplementary Information for

Oxygen availability and body mass modulate ectotherm responses to ocean warming

Murray I Duncan*, Fiorenza Micheli, Thomas H Boag, J. Andres Marquez, Hailey Deres, Curtis Deutsch, Erik A Sperling

*Corresponding author, Murray I. Duncan.
Email: murray.duncan@unisey.ac.sc

This PDF file includes:

Figures S1 to S4
Tables S1

Supplementary Figure S1: Linear models used to estimate mass scalers (slope) for standard metabolic rates (SMR, A) and critical oxygen partial pressure (Pcrit, C), linear model to estimate parameters of Arrhenius temperature effect on SMR (C) and quadratic polynomial model for temperature effect on Pcrit (D) for *Strongylocentrotus purpuratus*. Raw experimental data is indicated by purple points, best fit model is solid black line and 95% confidence intervals are shaded grey. Std. Er. is standard error, t-Val. is the t- statistic, p-val. Is the p-value, df is degrees of freedom, f-stat is the f statistic and R2 is r-squared. Source data are provided as a Source Data file.

SMR

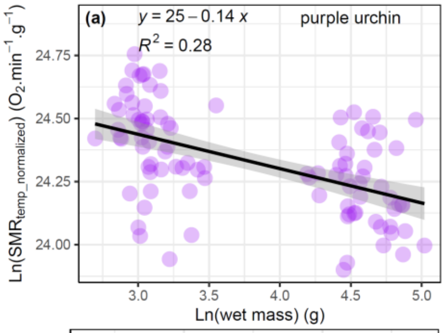

	Estimate	Std. Er.	t-val.	p-val.
**Intercept**	24.85	0.09	276.87	< 2e-16
**Slope**	-0.14	0.02	-6.10	2.2e-08
**df**	95.00			
**f-Stat**	37.28			
**R2**	0.27			

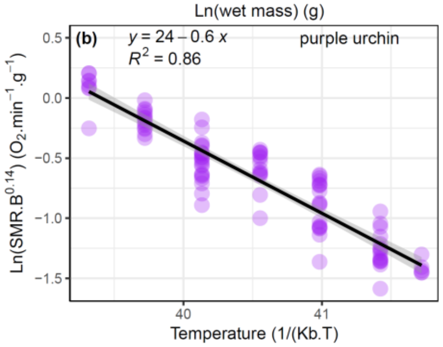

	Estimate	Std. Er.	t-val.	p-val.
**Intercept**	23.69	0.99	23.95	< 2e-16
**Slope**	-0.60	0.24	-24.63	< 2e-16
**df**	95.00			
**f-Stat**	606.8			
**R2**	0.86			

Pcrit

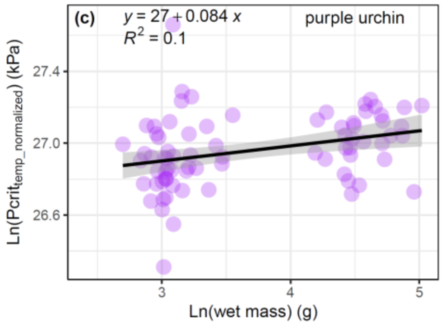

	Estimate	Std. Er.	t-val.	p-val.
**Intercept**	26.64	0.11	242.01	< 2e-16
**Slope**	0.08	0.03	2.87	0.00538
**df**	74.00			
**f-Stat**	8.224			
**R2**	0.09			

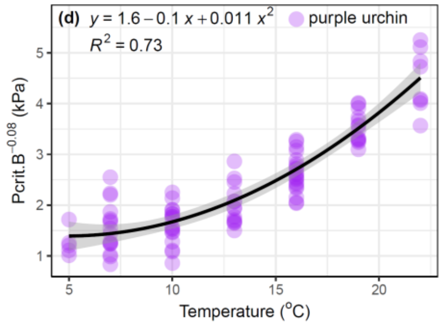

	Estimate	Std. Er.	t-val.	p-val.
**Intercept**	1.64	0.37	4.44	2.47e-05
**X1**	-0.10	0.06	-1.74	0.0859
**X2**	0.01	0.00	4.874	4.44e-06
**df**	94			
**f-Stat**	198.8			
**R2**	0.80			

Supplementary Figure S2: Linear models used to estimate mass scalers (slope) for standard metabolic rates (SMR, A) and critical oxygen partial pressure (Pcrit, C), linear model to estimate parameters of Arrhenius temperature effect on SMR (C) and linear model to estimate the parameters of general exponential model for temperature effect on Pcrit (D) for *Haliotis rufescens*. Raw experimental data is indicated by red points, best fit model is solid black line and 95% confidence intervals are shaded grey. Std. Er. is standard error, t-Val. is the t- statistic, p-val. Is the p-value, df is degrees of freedom, f-stat is the f statistic and R2 is r-squared. Source data are provided as a Source Data file.

SMR

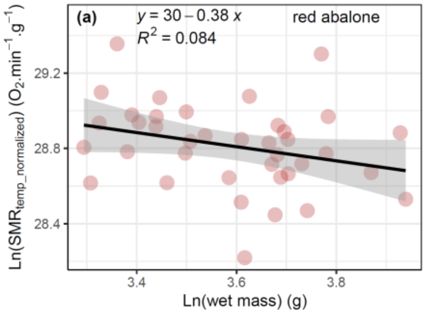

	Estimate	Std. Er.	t-val.	p-val.
**Intercept**	30.16	0.74	40.66	< 2e-16
**Slope**	-0.38	0.21	-1.82	0.0755
**df**	36			
**f-Stat**	3.30			
**R2**	0.06			

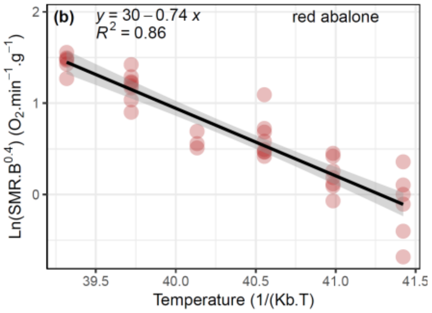

	Estimate	Std. Er.	t-val.	p-val.
**Intercept**	40.50	2.00	15.26	< 2e-16
**Slope**	-0.74	0.05	-14.93	< 2e-16
**df**	36			
**f-Stat**	222.8			
**R2**	0.86			

Pcrit

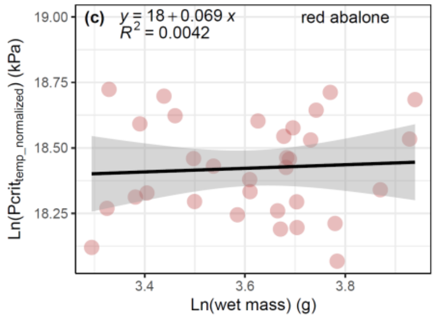

	Estimate	Std. Er.	t-val.	p-val.
**Intercept**	18.17	0.70	25.87	< 2e-16
**Slope**	0.07	0.19	0.35	0.726
**df**	30			
**f-Stat**	0.13			
**R2**	0.00			

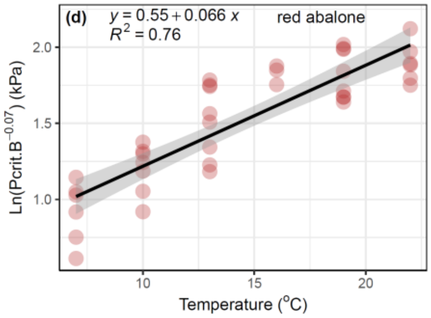

	Estimate	Std. Er.	t-val.	p-val.
**Intercept**	0.55	0.09	5.842	1.13e-06
**Slope**	0.07	0.01	10.701	9.86e-13
**df**	36			
**f-Stat**	114.5			
**R2**	0.75			

Estimating Φ_A' critical threshold. To estimate an Φ_A' threshold we plotted the distribution of every mean monthly Φ_A' value from geo-referenced occurrence locations and took the threshold as the 0.01 percentile and estimate a critical Φ_A' threshold of 0.928 for *H. rufescens* and 0.849 for *S. purpuratus* (Fig. S3).

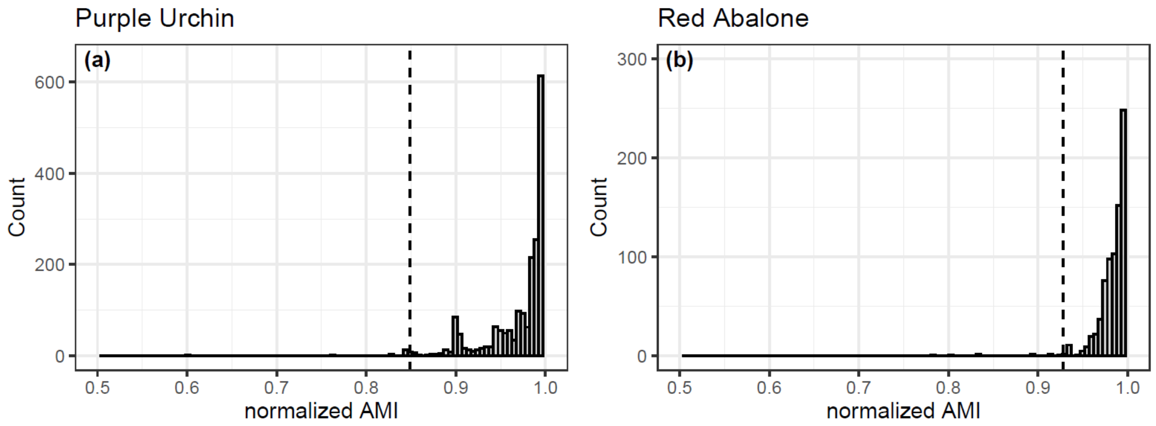

Supplementary figure S3. Critical Φ_A' determination (dashed line) for *S. purpuratus* (a) and *H. rufescens* (b) taken as the normalized absolute metabolic index (Φ_A') value above which includes 99% of mean monthly Φ_A' values organisms are currently exposed to in the wild. Source data are provided as a Source Data file.

Evaluating PO_{2crit} estimation robustness. We find close to a one-to-one relationship between PO_{2crit} quantified with the methods described in this study and PO_{2crit} quantified as the oxygen partial pressure where oxygen supply capacity (α_S) is maximized, following Seibel et al. 2021 (31) (Fig. S4). We take this as evidence that our technique to estimate PO_{2crit} in relation to standard metabolic rate is robust.

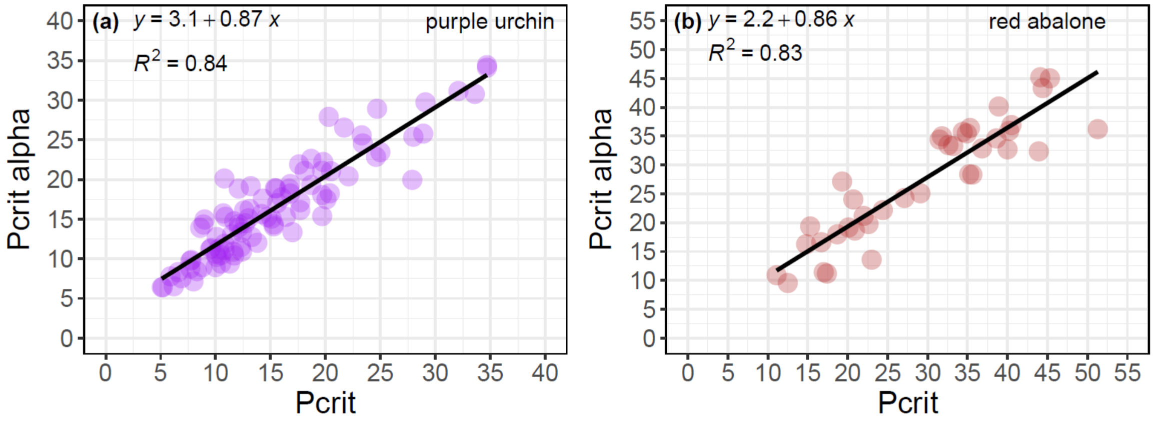

Supplementary figure S4. Linear relationships (solid black line) between the critical oxygen partial pressure (Pcrit) quantified using methods described in this study and an alternative method (Pcrit alpha) for *S. purpuratus* (purple) and *H. rufescens* (red). Source data are provided as a Source Data file.

Supplementary table S1. Checklist of information required to replicate respirometry studies following Killen et al. 2021 (*72*)

#	Category	Response
**Equipment, materials, and set-up**		
1	body mass at time of respirometry	Following a respirometry trial organisms were immediately frozen and later thawed to measure their wet mass, dried in a oven at 50 °C for ~ 24 hours to measure dry mass and then ashed to measure metabolic mass.
2	empty respirometer volume	two sized respirometers were used depending on the size of organism. The Large respirometer had a volume of 473.2 ml (16 oz) and the small respirometer had a volume of 177.4 ml (6 oz).
3	mixing device	water was continuously mixed via an in-line pump and circulation loop
4	respirometer volume to body mass ratio	respirometer volume:mass ratio ranged from 3.8 - 20.8 (information for each trial run is reported in the metadata).
5	material of tubing	clear thick wall vinyl tubing.
6	volume of tubing	volume of tubing in beg respirometers was 106.8 ml and volume of tubing for small respirometers was 97.6 ml.
7	volume of tubing included in analysis	confirmed
8	respirometer materials	polyethylene terephthalate plastic
9	oxygen probe type	Pyroscience OXSP5 sensor spots
10	sampling frequency of oxygen	Oxygen sampled every second throughout trial
11	oxygen probe placement	Oxygen probe placed in the circulation loop
12	flow rate/return to normoxia	Flushing flow rate was controlled via a one-way valve on the flush line. Flush flow rate was not directly measured but it was ensured chambers returned to 100% saturation following measurement periods
13	timing of flush/closed cycles	10-minute flush, 5 minute measure (10 minute measure for two trials at 7°C in metadata).
14	measurement exclusion start and end	one minute was excluded from the start and end of each measurement cycle resulting in a 3 min measurement period for metabolic rate calculation
15	frequency and calibration of probe	Oxygen probes was calibrated in 100% saturated water before each trial
16	software temperature compensation	Software temperature compensation was not used. In cases when the chiller turned on to maintain water during a measurement period the data were excluded
**Measurement conditions**		
17	trial temperature	Trial temperatures were either 5, 7, 10, 13, 16, 19 or 22 °C and included in the metadata
18	how temperature was controlled	Temperature was controlled with an Inkbird temperature controller connected to an aquarium heater and chiller
19	photoperiod during respirometry	All respirometry was carried out in the dark (water bath coolers lids closed)
20	water cleaning	Ambient water bath was constantly aerated with an aquarium air pump. Water was replaced after each trial
21	volume of ambient water bath	Ambient water bath volume was approximately 150 liters

22	minimum oxygen level reached	oxygen levels during intermittent flow metabolic rate measurements never fell below 85% saturation
23	chambers visually shielded	Yes, chambers were placed inside closed cooler box's and thus visually shielded from all disturbance
24	how many animals per trial	3, 4, 5, 6 or 7 animals were run per trial
25	animals shielded from each other	Animals were not shielded from each other during trials however trials were carried out in the dark and the study species naturally occur in groups
26	fasting period	Specimens were obtained from a cage in their natural environment from Monterey Abalone Company and placed directly into respirometers where they were given 24 hours to adjust to the novel environment which is considered part of the fasting period = minimum 24 hours
27	duration of all trials	Trials began on 17/07/202 and ended on 20/11/2020
28	acclimation time since capture	each specimen was given ~24 hours post transfer from wild to respirometry setup to acclimatize
**Background respiration**		
29	background respiration	Background respiration rates were measured in parallel in an empty container (alternated) throughout the duration of each trial.
30	how many background respiration slopes	Same as number of measurement slopes which is included in the metadata for each trial.
31	how were changes in background respiration modelled	They were not modelled, rather matched to corresponding measurement periods.
32	level of background respiration as a percentage	Background respiration as a percentage of organismal respiration is reported in the metadata.
33	method and frequency of system cleaning	Respirometers were cleaned with bleach twice throughout the experiment period when respirometer sizes were swapped. Water was replaced after every trial.
**Standard metabolic rate**		
34	time to metabolic rate measurements	Animals were inside respirometers for ~ 24 hours before metabolic rate measurements were considered
35	hours of metabolic rate measurements	Metabolic rate measurements were recorded for ~ 24 hours for each trial
36	state metabolic rate determination technique	Metabolic rate was taken as 0.1 quantile following Chabot et al (2016).
37	total number of slopes used to estimate SMR	Number of slopes used to estimate SMR ranged from 22 - 92, the majority trials (121/135) had more than 60 slope. The number of slopes per trial is included in the metadata
38	state whether any time periods were removed	Following an ~ 24 hour acclimation period intermittent flow respirometry (10 min flush, 5 min measure) was run for 20 - 24 hours = 80 - 96 potential measurements, We excluded measurements below an R2 threshold of 0.95 and also measurements associated with blanks in the upper 0.95 or lower 0.05 quantiles (chilling or heating device kicked on).
39	R2 thresholds	0.95 (0.9 for three samples at 7 °C)

40	proportion of outlier data removed	Most trial runs excluded less than 20% of oxygen consumption rates as outliers. Information for each trial is reported in metadata.
**Data handling and statistics**		
50	sample size	N = 97 (urchins), N = 38 (abalone)
51	state how oxygen uptake rates were calculated	Oxygen uptake rates were calculated following equation 3
52	confirm that volume (or mass) of animal was subtracted from resp volume	confirmed
53	specify whether body mass was accounted for	Oxygen uptake rates were converted to mass specific by dividing each rate by body mass and species specific allometric scaling exponents.
