# Supplementary material for: Oxygen availability and body mass modulate ectotherm responses to ocean warming
Source: Nat Commun. 2023 Jun 27;14:3811. doi: 10.1038/s41467-023-39438-w (PMC10300008; doi:10.1038/s41467-023-39438-w)
Supplement: Supplementary file 3 — Reporting Summary [file 41467_2023_39438_MOESM3_ESM.pdf]

nature portfolio

Corresponding author(s): Murray I Duncan

Last updated by author(s): 05/05/2023

Reporting Summary

Nature Portfolio wishes to improve the reproducibility of the work that we publish. This form provides structure for consistency and transparency in reporting. For further information on Nature Portfolio policies, see our Editorial Policies and the Editorial Policy Checklist.

Statistics

For all statistical analyses, confirm that the following items are present in the figure legend, table legend, main text, or Methods section.

n/a	Confirmed	
☐	☒	The exact sample size (*n*) for each experimental group/condition, given as a discrete number and unit of measurement
☐	☒	A statement on whether measurements were taken from distinct samples or whether the same sample was measured repeatedly
☐	☒	The statistical test(s) used AND whether they are one- or two-sided<br>*Only common tests should be described solely by name; describe more complex techniques in the Methods section.*
☐	☒	A description of all covariates tested
☐	☒	A description of any assumptions or corrections, such as tests of normality and adjustment for multiple comparisons
☐	☒	A full description of the statistical parameters including central tendency (e.g. means) or other basic estimates (e.g. regression coefficient) AND variation (e.g. standard deviation) or associated estimates of uncertainty (e.g. confidence intervals)
☐	☒	For null hypothesis testing, the test statistic (e.g. *F*, *t*, *r*) with confidence intervals, effect sizes, degrees of freedom and *P* value noted<br>*Give P values as exact values whenever suitable.*
☒	☐	For Bayesian analysis, information on the choice of priors and Markov chain Monte Carlo settings
☒	☐	For hierarchical and complex designs, identification of the appropriate level for tests and full reporting of outcomes
☒	☐	Estimates of effect sizes (e.g. Cohen's *d*, Pearson's *r*), indicating how they were calculated

Our web collection on statistics for biologists contains articles on many of the points above.

Software and code

Policy information about availability of computer code

Data collection	Oxygen levels in chambers were continuously recorded with a FireStingO2 fiber optic oxygen meter (FSO2-4, Pyro Science GmbH) connected to oxygen sensor spots (Pyroscience OXSP5) positioned in a circulation loop with bare optical fibers (SPFIB-BARE, Pyro Science GmbH) and logged with Pyro oxygen logger software
Data analysis	All analysis was performed in R programming language using version 4.2.0. The following packages were used in the analysis; RespR, respirometry, oce, ggspatial, raster, marmap, rnaturalearth, sf, viridis, tidyverse, ggpubr, gridExtra, robis, rgbif, reshape

For manuscripts utilizing custom algorithms or software that are central to the research but not yet described in published literature, software must be made available to editors and reviewers. We strongly encourage code deposition in a community repository (e.g. GitHub). See the Nature Portfolio guidelines for submitting code & software for further information.

Data

Policy information about availability of data

All manuscripts must include a data availability statement. This statement should provide the following information, where applicable:

- Accession codes, unique identifiers, or web links for publicly available datasets
- A description of any restrictions on data availability
- For clinical datasets or third party data, please ensure that the statement adheres to our policy

The raw respirometry data generated during experiments and the processed data used for the analyses in this study have been deposited in the Zenodo database accessible through the following DOI:10.5281/zenodo.7899786. Source data for figures is included as a supplementary file.

Human research participants

Policy information about studies involving human research participants and Sex and Gender in Research.

Reporting on sex and gender	No human research participants were used in this study.
Population characteristics	No human research participants were used in this study.
Recruitment	No participants were recruited for this study.
Ethics oversight	No ethics was required as no human participants were used in this study.

Note that full information on the approval of the study protocol must also be provided in the manuscript.

Field-specific reporting

Please select the one below that is the best fit for your research. If you are not sure, read the appropriate sections before making your selection.

☐ Life sciences ☐ Behavioural & social sciences ☒ Ecological, evolutionary & environmental sciences

For a reference copy of the document with all sections, see nature.com/documents/nr-reporting-summary-flat.pdf

Ecological, evolutionary & environmental sciences study design

All studies must disclose on these points even when the disclosure is negative.

Study description	We developed a model that integrates environmental oxygen, temperature, and body mass to predict habitat suitability for aquatic organisms. The parameters of this model are species-specific, calibrated with physiological measurements; basal metabolism (SMR) and critical oxygen level (PO2crit) across a temperature range. In this study we first generated the necessary physiological data for two species: purple urchin (Strongylocentrotus purpuratus) and red abalone (Haliotis rufescens). We used this data to calibrate parameters of our new model and then qualitatively compare its predictions of optimal and viable oceanic conditions with empirical evidence.<br><br>For the physiology experiments we quantified basal metabolism (SMR) and critical oxygen level (PO2crit) at seven temperatures (5,7,10,13,15,19,22 deg C). We used a specimen at only one temperature and replicated the temperature several times. we used a total of 97 purple urchin and 38 red abalone across the temperatures tested. We modelled the relationship of our dependent physiological variables as an Arrhenius function of temperature except for purple urchin PO2crit for which we used a more flexible quadratic polynomial to fit the data. We estimated mass scalers using power functions as is standard practice. Once model parameters were calibrated, we used them to predict optimal temperatures (highest values) for various hypothetical oxygen levels and body masses. We also obtained bottom temperature and oxygen data from an ocean model for the California current system (the species' core range) and predicted spatial habitat suitability for current and future (2100) climate scenarios.
Research sample	Organisms used for this study were benthic invertebrate's endemic to the California Current Ecosystem; purple urchin (Strongylocentrotus purpuratus) and red abalone (Haliotis rufescens). We wanted to work in the California current system due its variable temperature and oxygen regime and therefore selected model species that were easily attainable and distributed throughout the system. Specimens were obtained from Monterey Abalone Company, in Monterey close to where the experimental trials were carried. Specimens were housed in underwater cages as part of the companies aquaculture operation and fed a naturally diet of kelp. Batches of specimens were obtained prior to beginning each trial ensuring at least a 2-fold size difference was present. We made measurements for as many organisms as feasibly possible aiming for at least four replicates per temperature treatment (5,7,10,13,15,19,22). Ultimately we created the biggest PO2crit dataset in terms of temperature range and number of replicates for any species to date (Strongylocentrotus purpuratus; n = 97, wet mass range 14.8 – 151.3 g and Haliotis rufescens; n = 38, wet mass range 26.9 – 51.3 g) ensuring it represented population general population responses.
Sampling strategy	The sampling procedure involved making batches of physiological measurements at set temperatures sequentially. We did not pre-determine a sample size but rather took as many samples as feasibly possible ensuring test temperatures were replicated and a wide

	test temperature range (5 – 22 deg C). Collecting the respirometry data is time intensive and number of concurrent samples is constrained by equipment. Ultimately, we created the largest datasets of temperature-dependent hypoxia tolerance for any species (Strongylocentrotus purpuratus; n = 97, Haliotis rufescens; n = 38) and over the widest temperature range. We are confident this data adequately represents population trends.
Data collection	Respirometry trials were conducted in batches (equipment constrained) and at a single temperature (equipment designed operate at constant temperature). Procedure involved obtaining a batch of organisms from Monterey Abalone Company, placing them into respirometers at ambient temp, adjusting temperature to set experimental temps (5,7,10,13,16,19,22), allowing ~12 hours to acclimate, doing ~ 22 hours of intermittent flow respirometry where oxygen consumption was recorded, then closing the chamber completely until the organisms used all remaining oxygen and PO2crit is reached. All respirometry data were collected by the corresponding author.
Timing and spatial scale	We ran respirometry trials sequentially from 07-17-2020 until 11-20-2020. We collected data for as long a time as feasibly possible without any sampling gaps. All data were collected in Monterey at Hopkins Marine Station using organisms from Monterey Bay. This location is towards the center of both species' distributions.
Data exclusions	Data from all trial runs were used in the analysis unless oxygen failed to drawdown to zero during the PO2crit part of the trial which indicated a leak in the respirometer. This criterion was pre-established and such cases were rare.
Reproducibility	We replicated physiological trials for each species at each test temperature and combined data into a single analysis. Physiological measurements from comparable trials were similar. We included a check box in the supplement corresponding to recommendations of Killen et al. 2021 to ensure experimental set-up and results are reproducible. Physiological data is provided in the supplement and code is available from the authors. Trials were replicated thirty times across the test temperatures resulting in multiple replicates per species per temperature for the analysis.
Randomization	Prior to each trial a random selection of specimens were obtained from the aquaculture cage ensuring a variable size distribution. Specimens were then placed into randomly assigned respiromters for data collection. This process was repeated for each subsequent trial at each test temperature. Controlling for covariates was not relevant to this study as experiments were run in a tightly controlled environment, all specimens were treated identical with only body mass being variable which is included in the final model.
Blinding	Blinding was not possible in thus study as the two species could be distinguished by eye and the temperature of the trial and specimen body mass was required for the analysis. When analyzing physiological measurements, the researcher could not be blinded from this information. However, we analyzed our physiological data with an automated analytical script including only quantifiable input variables (temperature, mass, respirometer size, blank channel, start time, end time) minimizing interpretation and bias. The analysis script will reproduce physiological measurements from raw data files which are available.

Did the study involve field work? ☐ Yes ☒ No

Reporting for specific materials, systems and methods

We require information from authors about some types of materials, experimental systems and methods used in many studies. Here, indicate whether each material, system or method listed is relevant to your study. If you are not sure if a list item applies to your research, read the appropriate section before selecting a response.

Materials & experimental systems

n/a	Involved in the study
☒	☐ Antibodies
☒	☐ Eukaryotic cell lines
☒	☐ Palaeontology and archaeology
☐	☒ Animals and other organisms
☒	☐ Clinical data
☒	☐ Dual use research of concern

Methods

n/a	Involved in the study
☒	☐ ChIP-seq
☒	☐ Flow cytometry
☒	☐ MRI-based neuroimaging

Animals and other research organisms

Policy information about studies involving animals; ARRIVE guidelines recommended for reporting animal research, and Sex and Gender in Research

Laboratory animals	purple urchin (Strongylocentrotus purpuratus) and red abalone (Haliotis rufescens) obtained from a cage aquaculture farm in the region. The age of specimens was not known but all were adults.
Wild animals	no wild animals were used
Reporting on sex	Sex was not considered in this study. Purple urchins are externally indistinguishable between the sexes which made classifying specimens as either male or female unfeasible. Findings form this study apply to the populations as a whole.

Field-collected samples	no field collected samples were used
Ethics oversight	No ethical approval was required as organisms used were invertebrates and were not obtained from the wild.

Note that full information on the approval of the study protocol must also be provided in the manuscript.
