# Supplementary material for: Oxygen availability and body mass modulate ectotherm responses to ocean warming
Source: Nat Commun. 2023 Jun 27;14:3811. doi: 10.1038/s41467-023-39438-w (PMC10300008; doi:10.1038/s41467-023-39438-w)
Supplement: Supplementary file 2 — Peer Review File [file 41467_2023_39438_MOESM2_ESM.pdf]

nature portfolio

Peer Review File

Oxygen availability and body mass modulate ectotherm responses to ocean warming

Open Access This file is licensed under a Creative Commons Attribution 4.0 International License, which permits use, sharing, adaptation, distribution and reproduction in any medium or format, as long as you give appropriate credit to the original author(s) and the source, provide a link to the Creative Commons license, and indicate if changes were made. In the cases where the authors are anonymous, such as is the case for the reports of anonymous peer reviewers, author attribution should be to 'Anonymous Referee' followed by a clear attribution to the source work. The images or other third party material in this file are included in the article's Creative Commons license, unless indicated otherwise in a credit line to the material. If material is not included in the article's Creative Commons license and your intended use is not permitted by statutory regulation or exceeds the permitted use, you will need to obtain permission directly from the copyright holder. To view a copy of this license, visit http://creativecommons.org/licenses/by/4.0/.

REVIEWER COMMENTS</B>

Reviewer #1 (Remarks to the Author):

Dear authors and Editor,

I very much enjoyed reading "Oxygen availability and body mass modulate organismal responses to ocean warming". I think it is a well-designed and study with potential to greatly contribute to our ability to understand and predict species responses to climate change. I for one got very excited to try this framework for understanding the combined effects of warming and deoxygenation on the distribution of marine organisms.

Duncan et al expand the metabolic index framework (Deutsch et al 2020, Nature) to an absolute index. According to the authors, this allows quantification of the entire thermal envelope. The major benefit of this is that it can be used to project changes in abundance in across heterogenous environments with local hotpots of warming and deoxygenation—something recent studies have shown is important to consider (it also predicts why thermal optima should decline with size, which is also interesting). Hence, this study could be key for advancing the mechanistic basis of species distribution modelling in relation to climate change, which to my understanding is mostly correlative. However, I say "according to the authors" above because while I trust that is the case that the absolute index has the said advantages over the ratio that is the metabolic index, it is not intuitive to me why it is so. I think there is potential to describe the differences, benefits, and novelty of this absolute index more clearly in relation to the factorial index. This is because I think it has potential as a general framework for researchers that are not immediately familiar with eco-physiology but work with e.g., species distribution modelling.

Besides these potential issues in presentation, the authors provide a clear description of the experimental protocol (which seems standard based on the references they cite, though I am not an experimentalist myself) and software used to convert rates and fit models. Hence, I recommend only minor changes.

General comments
Lines 87-98: Here you first describe the benefits of using this absolute index over the factorial metabolic index. You say:
"[…] species' entire thermal envelope (both warm and cool edges) because of its factorial form when oxygen supply is governed by a single exponential functon(e.g., Fig. 2 in 26)."

To me it is not evident why this is. I understand that your index is a difference, and the other is a ratio, but the emerging differences that you point to that are due to this fact is not clear to me. Instead of referring to a specific figure in a different paper, would it be possible to try to illustrate why this?

Minor comments
1. There are some sentences that are quite long
a) First two sentences in intro quite long, at least one could be split up
b) Lines 415-419 could also be split up

2. Line 61: should it be both "rate" and "process" here?

3. Line 68: comma before "respectively"?

4. In Fig. 1, could the legend colors be matched more precisely with the figure?

5. Fig. 1. For consistency with text and equations, would it not make more sense to have labels PO_{2crit} and PO_{2crit\ max} as labels for colored areas instead of Pcrit and Pcrit(max)?

6. In Eq. 1 and on line 134, subscript d is used for standard metabolic rate but it should be capitalized, right?

7. I don’t really follow eq. 2:
S=α_S(T)PO_2B^σ (From Eq. 1)
α_S=α_{D(T)}/PO_{2crit(T)} (From line 144)
Then S can be written as (switched place to match text):
S=PO_2B^σ{(α}_{D(T)}/PO_{2crit(T)}).
But in Eq. 2 S is written as:
S=PO_2B^{δ-σ}{(α}_{D(T)}/PO_{2crit(T)}).
I.e., I don’t follow why it is B^{δ-σ} instead of B^σ. (And I also don’t know what the full stops in Eq. 2 refers to. Apologies if this is basic but I simply do not understand it).

8. Line 182: Should it be scalars instead of scalers? Or simply exponents, for consistency with how delta is described on line 517.

9. Line 144: Could you clarify why \alpha_{S(T)} is ratio between basal oxygen demand and the critical oxygen partial pressure?

10. Line 436: Do you view this as an extension of the metabolic theory of ecology (MTE), or in a more general way that it's a metabolic-based framework? I think the latter because you for instance do not invoke universal temperature and mass scaling. I think therefore confusion with the MTE should be avoided here.

11. Line 522: Was this really done first (“We first multiplied SMR data with its Arrhenius function to remove the effect of temperature and fit”)? Which value did you use for -E, if this was estimated in the next step (the log-log model of rate~(1/(k*T))?

12. Lines 522-526: Instead of correcting for mass and temperature separately, did you consider simply fitting a multiple regression? log(rate) ~ 0 + 1/(k*T) + log(mass), such that the coefficients become intercept (log(alpha)), activation energy and mass-exponent?

13. Line 530: Best to clarify: similar to what?

14. Line 550: I don’t think you need to insert Eq. 2 again here, probably a bit confusing since Eq. 5 comes right after.

15. Lines 575-578: I don’t really follow this. The goal was to get environmental data as close to the seafloor as possible, right? Why 200 meters? And if you had depth in the environmental data (else, how did you match it with the bathymetry), why not taking the deepest point? I’m probably just missing something obvious here)

16. Fig. 6. Is it possible to use a different color for “absence”? It’s not very visible on that background. I think the presences are more interesting to compare of course, but since you have absences mapped, they should be visible.

17. Ref no. 60 is now published (https://doi.org/10.1111/gcb.16067)

18. In the supplement, row 37, “of o.89”. Change to 0.89.

Reviewer #2 (Remarks to the Author):

The manuscript “Oxygen availability and body mass modulate organismal responses to ocean warming” developed an absolute aerobic scope index to investigate the temperature, oxygen, and mass dependence of aerobic metabolism of two aquatic ectotherms, and then uses the index to predict the impacts of climate change through its effects on water temperature and oxygen levels. I find great value in the authors’ effort to provide a mechanistic model of the niche’s temperature and oxygen dimensions. I think the motivation of the manuscript is clear and the model is well explained and justified.

The index seems to be useful at extrapolating aerobic scopes from observed thermal responses of

Pcrit. Indeed, predicted and observed Topts suggest that the model accurately predicts the thermal dependence of metabolism of both S. purpuratus and H. rufescens. However, the extrapolation of these thermal responses to the ecological niche seems problematic. Thus, although the predictions for S. purpuratus fit remarkably well with its occurrence data, the model overestimates the suitable range of H. rufescens. In fact, just looking at the model predictions, it seems that H. rufescens is more generalist (in terms of oxygen and temperature dependence) than S. purpuratus, whereas occurrence data exhibit the exact opposite pattern.
The mismatch between the predicted aerobic scope and observed occurrence data makes me think that specie's current geographical distribution is not ultimately determined by temperature and oxygen relationships; instead, other ecological factors such as nutrient availability or species interactions may be more critical. Since the index focuses on specific abiotic components disregarding, e.g., biotic interactions, the spatial and temporal projections of the niche could be inaccurate, which limits its capacity to make predictions for the species' responses to climate change.

The manuscript does not explicitly address the predictive capacity of the index and does not provide a discussion about its limitations when forecasting the impacts of climate change. I think the authors should first investigate the capacity of the index to predict the current geographical ranges or niches of each species, and then use this to assess the likelihood of the climate change projections.

Line comments:

Lines 88-89: This idea is difficult to visualize. If I understood well, the argument here is that the exponential function shows a monotonic increase rather than a unimodal curve with an optimal temperature that can be used to delimit both the upper and lower thermal limits. I think Fig 2 in Deutsch et al. (2020) doesn't clearly show that the factorial aerobic scope cannot delimit both warm and cool edges since it represents a geographical projection rather than the variation of the index in relation to temperature.

Line 147: Then, contrary to the factorial index, the absolute form has units of mmolO2 h-1?

Line 157: "however" here is misleading since normalization does not avoid potential errors arising from the assumption that oxygen supply increases linearly with oxygen availability.

Lines 183: Are these allometric (mass) scalers or temperature coefficients?

Lines 289-291: The index overestimates the viable habitat for H. rufescens and may therefore produce inaccurate predictions of its responses to climate change.

Lines 293-296: Suitability is overestimated at both cold (<5ºC) and warm (>15ºC) temperatures, so lacking measurements at the coldest temperature does not explain the overall mismatch between predicted and observed niches.

Lines 409-410: The temperature-oxygen niche of H. rufescens is more limited than that predicted by the model, it would be necessary to assess whether these reductions and fractures in the geographical distribution will affect the existing populations.

Lines 518: Boltzmann's constant has units eV/K.

We would like to thank the reviewers for their thoughtful comments that we believe have all been addressed adequately. The suggestions have improved the manuscript. Specifically, reviewer 1 highlights that we need to explain the development of the absolute metabolic index more clearly and reviewer 2 highlights that we need to address the pitfalls of the absolute metabolic index better and not over sell it. These main comments have made the manuscript more accessible to a general audience and have better grounded it, while still highlighting the novel information this index elucidates.

Reviewer comments are included below with responses highlighted in bold while changes to the document are indicated in track changes. Two additional introductory figures (Fig. 1, A, B) have been included to graphically illustrate the benefit of this proposed index. We have also made the maps bigger to better illustrate the good fits for contemporary modelled distributions. Two additional supplementary figures have been included in the supplementary information to comply with Nature Communications reporting guidelines. A reproducible script to go along with the data is being compiled for an online depositary should the manuscript be accepted.

REVIEWER COMMENTS

Reviewer #1 (Remarks to the Author):

Dear authors and Editor,

I very much enjoyed reading “Oxygen availability and body mass modulate organismal responses to ocean warming”. I think it is a well-designed and study with potential to greatly contribute to our ability to understand and predict species responses to climate change. I for one got very excited to try this framework for understanding the combined effects of warming and deoxygenation on the distribution of marine organisms.

Duncan et al expand the metabolic index framework (Deutsch et al 2020, Nature) to an absolute index. According to the authors, this allows quantification of the entire thermal envelope. The major benefit of this is that it can be used to project changes in abundance in across heterogenous environments with local hotpots of warming and deoxygenation—something recent studies have shown is important to consider (it also predicts why thermal optima should decline with size, which is also interesting). Hence, this study could be key for advancing the mechanistic basis of species distribution modelling in relation to climate change, which to my understanding is mostly correlative. However, I say “according to the authors” above because while I trust that is the case that the absolute index has the said advantages over the ratio that is the metabolic index, it is not intuitive to me why it is so. I think there is potential to describe the

differences, benefits, and novelty of this absolute index more clearly in relation to the factorial index. This is because I think it has potential as a general framework for researchers that are not immediately familiar with eco-physiology but work with e.g., species distribution modelling.

Besides these potential issues in presentation, the authors provide a clear description of the experimental protocol (which seems standard based on the references they cite, though I am not an experimentalist myself) and software used to convert rates and fit models. Hence, I recommend only minor changes.

General comments
Lines 87-98: Here you first describe the benefits of using this absolute index over the factorial metabolic index. You say:
"[…] species' entire thermal envelope (both warm and cool edges) because of its factorial form when oxygen supply is governed by a single exponential functon(e.g., Fig. 2 in 26)."

To me it is not evident why this is. I understand that your index is a difference, and the other is a ratio, but the emerging differences that you point to that are due to this fact is not clear to me. Instead of referring to a specific figure in a different paper, would it be possible to try to illustrate why this?

We have updated the text here to try and make a more intuitive understanding of this concept. Additionally, we have provided two new theoretical figures that graphically depict the differences when expressing S/D vs S – D (Fig. 1 A, B)

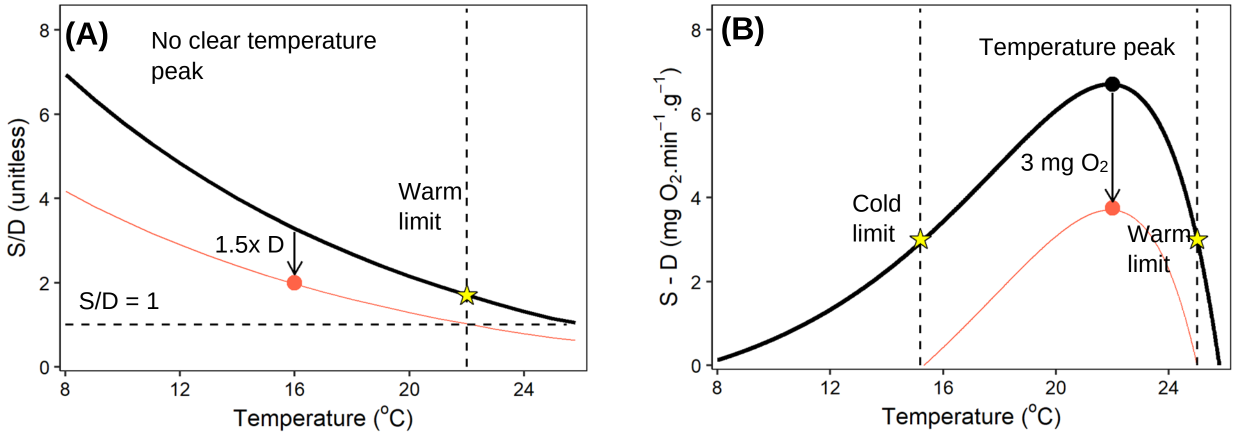

Figure 1. Shape of thermal relationships for different expressions of an organism's oxygen balance in saturated water. A) When the balance between oxygen supply (S) and demand (D) is expressed as a ratio (Solid black line) no clear temperature peak is identified if demand is a single monotonic function of temperature and only a single thermal limit can be identified (star). Note that the relationship between demand and temperature may not always be monotonic with temperature [28,30,31]. Thermal optima arise from the multi-step nature of O_2 supply, which can be readily reproduced by the factorial form of the metabolic index[31], but both the absolute and

factorial forms shown here assume a single temperature-dependent supply function. B) The same data expressed as the difference between S and D (solid black line) often yields a thermal peak and both warm and cold thermal limits can be estimated (stars). When additional energetic processes are performed the available oxygen balance decreases (red lines) in A and B. If these energetic processes are vital for performance a species' thermal limits are defined as those temperatures where oxygen supply falls to match elevated oxygen demand (equal to a value of one in A and zero in B).The oxygen requirement of these additional processes can be quantified and removed from the total oxygen budget in B but must be measured in relation to demand in A, which is challenging if thermal sensitivities differ (i.e. additional energetic process does not equal 1.5 x basal demand across whole temperature range).

Minor comments

1. There are some sentences that are quite long

a) First two sentences in intro quite long, at least one could be split up

1st sentence of the introduction has been split into two.

2nd sentence of the introduction has been shortened.

b) Lines 415-419 could also be split up

Sentence has been broken up and rewritten for clarity

2. Line 61: should it be both "rate" and "process" here?

"Rate" has been deleted

3. Line 68: comma before "respectively"?

Comma inserted

4. In Fig. 1, could the legend colors be matched more precisely with the figure?

Figure adjusted accordingly

5. Fig. 1. For consistency with text and equations, would it not make more sense to have labels PO_{2crit} and PO_{2crit\ max} as labels for colored areas instead of Pcrit and Pcrit(max)?

Figure adjusted accordingly

6. In Eq. 1 and on line 134, subscript d is used for standard metabolic rate but it should be capitalized, right?

Subscripts have been capitalized throughout for both supply (α_S) and demand (α_D)

7. I don’t really follow eq. 2:
S=α_S(T)PO_2B^σ (From Eq. 1)

α_S=α_{D(T)}/PO_{2crit(T)} (From line 144)

Then S can be written as (switched place to match text):
S=PO_2B^σ{(α}_{D(T)}/PO_{2crit(T)}).
But in Eq. 2 S is written as:
S=PO_2B^{δ-σ}{(α}_{D(T)}/PO_{2crit(T)}).

I.e., I don’t follow why it is B^{δ-σ} instead of B^σ. (And I also don’t know what the full stops in Eq. 2 refers to. Apologies if this is basic but I simply do not understand it).

Thank you for pointing this out. There was slight error when writing down equation 2. The mass scaling exponent for supply (σ, eqn. 1) is equivalent to the difference between mass scaling exponents for demand and PO2crit (δ – ε). This has been updated throughout and doesn’t change anything. Additional text added to clarify.

Full stops refer to multiplication between terms.

8. Line 182: Should it be scalars instead of scalers? Or simply exponents, for consistency with how delta is described on line 517.

Changed to “exponents”

9. Line 144: Could you clarify why \alpha_{S(T)} is ratio between basal oxygen demand and the critical oxygen partial pressure?

For an organism of a single mass at a single temperature the absolute metabolic index is:

$\Phi_A = S - D = \alpha_S.PO_2 - \alpha_D$.

If environmental oxygen drops to the organisms PO_{2crit} level, then supply = demand and therefore:

$\Phi_A = 0 = \alpha_S.PO_{2crit} - \alpha_D$.

Rearranging this equation is:

$\alpha_S = \alpha_D / PO_{2crit}$

The effect of mass on supply capacity (B^{σ}) is therefore equal to the ratio of the mass effect on demand and PO_{2crit} ($B^{\delta}/B^{\varepsilon}$):

$\alpha_S . B^{\sigma} = \alpha_D . B^{\delta} / PO_{2crit} . B^{\varepsilon}$

The effect of temperature on supply capacity (T) is therefore equal to the ratio of demand and PO_{2crit} given the prevailing temperature.

$\alpha_{S.(T)} . B^{\sigma} = \alpha_{D.(T)} . B^{\delta} / PO_{2crit.(T)} . B^{\varepsilon}$

Thus, total oxygen supply (S) is equal to oxygen supply capacity (considering mass and temperature effects) multiplied with environmental oxygen which is how we got to equation 2.

Text has been updated for clarity.

10. Line 436: Do you view this as an extension of the metabolic theory of ecology (MTE), or in a more general way that it's a metabolic-based framework? I think the latter because you for instance do not invoke universal temperature and mass scaling. I think therefore confusion with the MTE should be avoided here.

Reference to MTE has been deleted without sentence losing meaning

11. Line 522: Was this really done first ("We first multiplied SMR data with its Arrhenius function to remove the effect of temperature and fit")? Which value did you use for -E, if this was estimated in the next step (the log-log model of rate~(1/(k*T))?

Yes – we first estimated the Arrhenius function from log(rate) ~ (1/kT) + log(intercept) and temperature standardized by dividing rate data by this Arrhenius function following Deutsch et al. 2015. We only used the temperature range where data followed Arrhenius relationships closely. We then used all temperature standardized data to estimate mass scaling exponents. We then used these scaling exponents to mass standardize rate data and fit tighter, sometimes more flexible temperature relationship models.

12. Lines 522-526: Instead of correcting for mass and temperature separately, did you consider simply fitting a multiple regression? log(rate) ~ 0 + 1/(k*T) + log(mass), such that the coefficients become intercept (log(alpha)), activation energy and mass-exponent?

We estimated mass scaling exponents and temperature relationships separately. This was to be consistent with original metabolic index methods and because temperature data did not always follow an exponential function (i.e. PO2crit for purple urchin).

13. Line 530: Best to clarify: similar to what?

Clarified – “We followed the same approach to SMR for modelling PO2crit data”

14. Line 550: I don’t think you need to insert Eq. 2 again here, probably a bit confusing since Eq. 5 comes right after.

Equation deleted

15. Lines 575-578: I don’t really follow this. The goal was to get environmental data as close to the seafloor as possible, right? Why 200 meters? And if you had depth in the environmental data (else, how did you match it with the bathymetry), why not taking the deepest point? I’m probably just missing something obvious here)

The ocean model has 21 depth bins ten meters apart with a max depth of 200 meters below sea level. Both study organisms prefer shallower depths less than -100m but have been recorded at deeper depths ~ 150 meters. We have therefore adjusted the depth threshold in the analysis to 150 meters to match the maximum depth of both species.

Because the grid size of the ocean model was fairly large, in places where the continental shelf is steep the deepest grid cell covers a spatial area with large depth difference. In some areas (e.g. a sea mount) the seafloor depth at the top of the mount may be closer to a depth layer of the ocean model that is not the deepest. We wanted to ensure that each seafloor depth value of the bathymetry dataset was matched with environmental data from the closest depth layer of the ocean model.

Text in this section has been updated to improve clarity

16. Fig. 6. Is it possible to use a different color for “absence”? It’s not very visible on that background. I think the presences are more interesting to compare of course, but since you have absences mapped, they should be visible.

Absences have been removed from maps to focus on presences only. Figure updated.

17. Ref no. 60 is now published (https://doi.org/10.1111/gcb.16067)

Reference is changed (note; not highlighted in track changes due complications with reference manager).

18. In the supplement, row 37, “of o.89”. Change to 0.89.

Changed to “0.89”

Reviewer #2 (Remarks to the Author):

The manuscript “Oxygen availability and body mass modulate organismal responses to ocean warming” developed an absolute aerobic scope index to investigate the temperature, oxygen, and mass dependence of aerobic metabolism of two aquatic ectotherms, and then uses the index to predict the impacts of climate change through its effects on water temperature and oxygen levels. I find great value in the authors’ effort to provide a mechanistic model of the niche’s temperature and oxygen dimensions. I think the motivation of the manuscript is clear and the model is well explained and justified.

The index seems to be useful at extrapolating aerobic scopes from observed thermal responses of Pcrit. Indeed, predicted and observed Topts suggest that the model accurately predicts the thermal dependence of metabolism of both S. purpuratus and H. rufescens. However, the extrapolation of these thermal responses to the ecological niche seems problematic. Thus, although the predictions for S. purpuratus fit remarkably well with its occurrence data, the model overestimates the suitable range of H. rufescens. In fact, just looking at the model predictions, it seems that H. rufescens is more generalist (in terms of oxygen and temperature dependence) than S. purpuratus, whereas occurrence data exhibit the exact opposite pattern.
The mismatch between the predicted aerobic scope and observed occurrence data makes me think that specie’s current geographical distribution is not ultimately determined by temperature and oxygen relationships; instead, other ecological factors such as nutrient availability or species interactions may be more critical. Since the index focuses on specific abiotic components disregarding, e.g., biotic interactions, the spatial and temporal projections of the niche could be inaccurate, which limits its capacity to make predictions for the species’ responses to climate change.

We have added text through indicating that the fit is tighter for *S. purpuratus* compared to *H. rufescens* to better highlight the limitations of the index.

Specifically: additional sentence at end of section 2.3:

In our analyses, the agreement between species occurrences and Φ_A' is tighter for *S. purpuratus* compared to *H. rufescens*, which may result in projecting suitable habitat for *H. rufescens’* in areas where they do not naturally occur. As discussed below, such misfit can result from the accuracy of the primary model inputs, namely 1) experimental respirometry data, 2) modeled or measured seafloor oxygen and temperature conditions, and 3) biogeographic occurrence data.

the end of 2.4:

However, the certainty of predictions needs to be considered within the context of tightness of fit between Φ_A' habitat suitability and occurrences. The index overestimates viable habitat for *H. rufescens* (Fig. 5 and Fig. 6 insert) and hence may overestimate the species’ contemporary distribution and future climate mediated distribution contractions.

and included a caveat paragraph in the discussion pasted below:

While the Φ_A framework presented here for *S. purpuratus* and *H. rufescens* elucidates thermal optima, provides a mechanism behind the oxygen and mass dependence of thermal optima, and delimits both species' contemporary biogeography, it is not without important caveats. Only two invertebrate species were tested and whether the Φ_A framework generalizes to other taxa is unknown. Even among just these two taxa, metabolic niche predictions appear more accurate for *S. purpuratus* compared to *H. rufescens*, and there are multiple possible reasons for this difference. This poorer fit for *H. rufescens* may be because we did not measure physiological traits at the coldest temperature (5 °C) for *H. rufescens* and hence modelled a more monotonic thermal relationship for PO_{2crit} data, resulting in broader Φ_A' curves per oxygen level and potentially erroneous suitable Φ_A' predictions at cold temperatures (< 7 °C). We also found geographic bias for this species in the OBIS occurrence data we used, with few documented occurrences of *H. rufescens* recorded in the warmer Mexican ocean despite the species occurring there [51]. In fact, *H. rufescens* had less than half the geo-referenced occurrence points in OBIS as *S. purpuratus*, and both species showed large geographic areas where the species currently exists but is simply not recorded in OBIS. Like all modeling approaches, Φ_A' is sensitive to data inputs, and the predictive power for a given species will continue to improve as the three main data inputs—biogeographic occurrences, species-specific physiological parameters, and measured or modeled oceanographic conditions—continue to be better documented. A final plausible explanation is that the contemporary distribution of *H. rufescens* is constrained by additional factors other than temperature and oxygen availability, such as resource competition, predation or life history and habitat specialization [71,72]. Forecasted future distribution changes based solely on environmental variables therefore need to be considered in the context of over predicting contemporary biogeographic distribution and predicted future range contractions.

The manuscript does not explicitly address the predictive capacity of the index and does not provide a discussion about its limitations when forecasting the impacts of climate change. I think the authors should first investigate the capacity of the index to predict the current geographical ranges or niches of each species, and then use this to assess the likelihood of the climate change projections.

We have added a large caveat section highlighting that *H. rufescens* fit is poorer and to consider this when evaluating future predictions i.e. if contemporary ranges are over estimated then future range contractions may appear exaggerated as the model is losing areas where the species does not actually exist. We have also discussed some of the possible data-driven reasons why the fit may be poorer, specifically highlighting the relatively lack of occurrence data in OBIS compared to *S. purpuratus.*

Line comments:

Lines 88-89: This idea is difficult to visualize. If I understood well, the argument here is that the exponential function shows a monotonic increase rather than a unimodal curve

with an optimal temperature that can be used to delimit both the upper and lower thermal limits.

Yes, that is what we are trying to say. We have updated text in this section as per another reviewers request and included two additional figures (Fig 1. A, B) that visually depict what we're trying to say.

I think Fig 2 in Deutsch et al. (2020) doesn't clearly show that the factorial aerobic scope cannot delimit both warm and cool edges since it represents a geographical projection rather than the variation of the index in relation to temperature.

Fig 2 in Deutsch 2020 shows three metabolic index calibrations and projections over geographic space (north to south and depth). Inputs for the projection are environmental temperature and oxygen. At the surface where oxygen is fully saturated the index increases as temperatures get colder at poles (a, b) or warmer towards the tropics (c) depending on the slope of the monotonic exponential function in the index. Thus, low values of the metabolic index can delimit one side of the species north – south distribution but it doesn't explain why it is absent in areas with vary high values of the index on the other side of their distribution. In other words, why doesn't the northern shrimp range (Deutsch et al., 2020 Fig. 2A) extend all the way to the north pole?

Line 147: Then, contrary to the factorial index, the absolute form has units of mmolO2 h-1?

Yes, rather than a unitless values the absolute form's is equal to the oxygen consumption units used in the respirometry experiments (i.e. oxygen consumption rate per unit time).

Line 157: "however" here is misleading since normalization does not avoid potential errors arising from the assumption that oxygen supply increases linearly with oxygen availability.

"however" has been deleted.

Since increased blood oxygen affinity from hypoxia acclimation can compensate oxygen supply constraints, we are rather interested in how the temperature for maximum oxygen supply changes with hypoxia – rather than the initial constraint on supply.

Lines 183: Are these allometric (mass) scalers or temperature coefficients?

Allometric mass scaling exponents – text has been updated to clarify

Lines 289-291: The index overestimates the viable habitat for H. rufescens and may

therefore produce inaccurate predictions of its responses to climate change.

Additional sentences have been included at the end of the first paragraph in section 2.4 highlighting that the looser fit for *H. rufescens* may result in overestimating contemporary ranges and underestimating future range contractions, which needs to be considered.

Lines 293-296: Suitability is overestimated at both cold (<5ºC) and warm (>15ºC) temperatures, so lacking measurements at the coldest temperature does not explain the overall mismatch between predicted and observed niches.

This information has been moved to the discussion and incorporated as part of a caveat section on our modelling approach as suggested above.

Lines 409-410: The temperature-oxygen niche of H. rufescens is more limited than that predicted by the model, it would be necessary to assess whether these reductions and fractures in the geographical distribution will affect the existing populations.

We have included a caveat section in the discussion on this model overprediction and highlighted that this must be considered when evaluating future forecasts throughout text.

Lines 518: Boltzmann’s constant has units eV/K

Units corrected

REVIEWER COMMENTS

Reviewer #1 (Remarks to the Author):

Hi, thanks for this nice review. I think the new figure is very good, and I also think all the other minor edits made it more accessible to a general audience. I have one comment left on my comment #11 in the first review:

11. Line 522: Was this really done first ("We first multiplied SMR data with its Arrhenius function to remove the effect of temperature and fit")? Which value did you use for -E, if this was estimated in the next step (the log-log model of rate~(1/(k*T))?

- Yes – we first estimated the Arrhenius function from log(rate) ~ (1/kT) + log(intercept) and temperature standardized by dividing rate data by this Arrhenius function following Deutsch et al. 2015. We only used the temperature range where data followed Arrhenius relationships closely. We then used all temperature standardized data to estimate mass scaling exponents. We then used these scaling exponents to mass standardize rate data and fit tighter, sometimes more flexible temperature relationship models.

- Here in the reply you say you divide with the Arrhenius functon(which I think is correct), but in the revised Word article file (line 647) you say you multiply with the Arrhenius function. Can you make sure you did the correct thing and that the text matches that?

Reviewer #2 (Remarks to the Author):

I have reviewed again the manuscript "Oxygen availability and body mass modulate organismal responses to ocean warming". The authors have addressed all of my suggestions and comments adequately. The manuscript now presents the rationale for developing an absolute version of the metabolic index in a much clearer way. In addition, the Discussion now addresses the limitations of the geographical and niche projections of the metabolic index.

This is a very interesting paper and an important contribution towards gaining a mechanistic understanding of the organismal responses to climate change.

Reviewer #3 (Remarks to the Author):

I was asked to review the experimental (respirometry) methods of this manuscript, which had already been reviewed in full by other reviewers who were not experts in the experimental technique but in other aspects of the study. Based on the available information (descriptions of the respirometry trials in the methods section and presentation of results, but no raw respirometry data or traces), it sounds like experiments were carried out well and with appropriate consideration to the important aspects of respirometry, such as respirometry chamber and tubing materials (especially critical when doing hypoxia (Pcrit) trials), fasting period before respirometry is initiated (24 h here), and respirometry chamber to organism size (volume) ratio (detailed in Table S1). The latter ratio was 3.8 to 20.8 here, with 3.8 being past the lower end of the 10 to 50 that is usually recommended. However, the small ratio would only have been an issue for the very largest animals, and I am not overly concerned about it.

One thing about the experimental protocol that I found a bit unfortunate in the context of forecasting species responses to environmental conditions in the wild, is that all measurements of SMR and Pcrit – the two key trait that are used to calculate the metabolic index, which this study is all about – were made after acute exposure to the different test temperatures; temperature was changed to the target temperature at 1'C/h, after animals were given 24 h to settle before respirometry was performed over the following 24 h. It is well known that acute thermal exposure has a stronger effect on physiological rates such as SMR and Pcrit, compared to if the organism had been acclimated (for a few weeks) to each test temperature. This means that the thermal

sensitivities used to calculate the metabolic index are most likely overestimated compared to what they would be in nature – that is, the spatial mapping and predictions of viable habitat for the two species using the metabolic index (Figs. 6 and 7) are likely incorrect or at least imprecise. The authors do mention on line 470 that:

"Despite the nature of future predictions being inherently imprecise as they do not consider higher spatial or temporal environmental variability or mechanisms that confer resilience such as acclimation and generational adaptation, these future distribution forecasts can serve as a comparative tool to identify what species and areas are relatively vulnerable to warming and deoxygenation"

This indicates that they are aware of the issue with acute vs. chronic (acclimation) thermal responses, but do not mention it explicitly.

Another minor thing I took note of was that the duration of the closed (measurement) and flushing periods of the intermittent-flow respirometry technique was apparently always kept the same (5 min flushing, 10 min closed; detailed in table S1) across the wide range of experimental test temperatures (5 to 22'C). As the animals would have respired faster at the higher temperatures (as the presented data also shows), oxygen levels inside the respirometry chambers would have dropped (much) more at the higher vs. lower temperatures. I would have recommended that at least close durations were adjusted based on the trial temperature, so the drop in oxygen remained similar at the various temperatures. The authors detail in Table S1 that oxygen levels inside the respirometry chambers never fell below 85% air saturation at a given temperature, which is still high, so presumably the fixed closed duration has not affected estimates of SMR, as 85% air saturation is above Pcrit for both species, although red abalone does show a strong sensitivity to hypoxia at 50% air saturation in terms of the optimal temperature of the metabolic index (Fig. 5B).

Overall – without having seen the raw respirometry data – the experiments themselves appear to be well conducted, although the ecological relevance of the produced data and their use in predicting habitat suitability are questionable, given the acute nature of the lab thermal exposures vs. the presumably more stable (acclimated) thermal environment in nature.

We would like to thank the reviewers for their thoughtful comments that we believe have all been addressed adequately. Reviewer comments are included below with responses highlighted in bold while changes to the document are indicated in track changes. We have included a new reference (76) that is not in track changes due to formatting issues with reference manager software. A reproducible script to go along with the analysis, raw experimental data and metadata is being compiled for an online repository should the manuscript be accepted. We have included the respirometry data as per reviewer 3's request and will upload it to a repository with scripts if the manuscript is accepted.

REVIEWER COMMENTS

Reviewer #1 (Remarks to the Author):

Hi, thanks for this nice review. I think the new figure is very good, and I also think all the other minor edits made it more accessible to a general audience. I have one comment left on my comment #11 in the first review:

11. Line 522: Was this really done first ("We first multiplied SMR data with its Arrhenius function to remove the effect of temperature and fit")? Which value did you use for -E, if this was estimated in the next step (the log-log model of rate~(1/(k*T))?

- Yes – we first estimated the Arrhenius function from log(rate) ~ (1/kT) + log(intercept) and temperature standardized by dividing rate data by this Arrhenius function following Deutsch et al. 2015. We only used the temperature range where data followed Arrhenius relationships closely. We then used all temperature standardized data to estimate mass scaling exponents. We then used these scaling exponents to mass standardize rate data and fit tighter, sometimes more flexible temperature relationship models.

- Here in the reply you say you divide with the Arrhenius functon(which I think is correct), but in the revised Word article file (line 647) you say you multiply with the Arrhenius function. Can you make sure you did the correct thing and that the text matches that?

We had "-*E*" as the temperature sensitivity term in the Arrhenius equation (equation 4) to be consistent with previous iterations (Brown 2004). Therefore, multiplying by this Arrhenius function is the same as dividing by the Arrhenius function when temperature sensitivity is "*E*". We have adjusted the text and equation to reflect the second approach as it is more intuitive.

Reviewer #2 (Remarks to the Author):

I have reviewed again the manuscript “Oxygen availability and body mass modulate organismal responses to ocean warming”. The authors have addressed all of my suggestions and comments adequately. The manuscript now presents the rationale for developing an absolute version of the metabolic index in a much clearer way. In addition, the Discussion now addresses the limitations of the geographical and niche projections of the metabolic index.

This is a very interesting paper and an important contribution towards gaining a mechanistic understanding of the organismal responses to climate change.

Thank you for your valuable review that improved the manuscript.

Reviewer #3 (Remarks to the Author):

I was asked to review the experimental (respirometry) methods of this manuscript, which had already been reviewed in full by other reviewers who were not experts in the experimental technique but in other aspects of the study. Based on the available information (descriptions of the respirometry trials in the methods section and presentation of results, but no raw respirometry data or traces), it sounds like experiments were carried out well and with appropriate consideration to the important aspects of respirometry, such as respirometry chamber and tubing materials (especially critical when doing hypoxia (Pcrit) trials), fasting period before respirometry is initiated (24 h here), and respirometry chamber to organism size (volume) ratio (detailed in Table S1). The latter ratio was 3.8 to 20.8 here, with 3.8 being past the lower end of the 10 to 50 that is usually recommended. However, the small ratio would only have been an issue for the very largest animals, and I am not overly concerned about it.

One thing about the experimental protocol that I found a bit unfortunate in the context of forecasting species responses to environmental conditions in the wild, is that all measurements of SMR and Pcrit – the two key trait that are used to calculate the metabolic index, which this study is all about – were made after acute exposure to the different test temperatures; temperature was changed to the target temperature at 1’C/h, after animals were given 24 h to settle before respirometry was performed over the following 24 h. It is well known that acute thermal exposure has a stronger effect on physiological rates such as SMR and Pcrit, compared to if the organism had been acclimated (for a few weeks) to each test temperature. This means that the thermal sensitivities used to calculate the metabolic index are most likely overestimated compared to what they would be in nature – that is, the spatial mapping and predictions of viable habitat for the two species using the metabolic index (Figs. 6 and 7) are likely incorrect or at least imprecise. The authors do mention on line 470 that:

“Despite the nature of future predictions being inherently imprecise as they do not consider higher spatial or temporal environmental variability or mechanisms that confer resilience such as acclimation and generational adaptation, these future distribution forecasts can serve as a comparative tool to identify what species and areas are relatively vulnerable to warming and deoxygenation”

This indicates that they are aware of the issue with acute vs. chronic (acclimation) thermal responses, but do not mention it explicitly.

We have included text to explicitly mention the words chronic/acute exposure and acclimation on lines 471 – 483 and stated that this study only measured acute exposures. The point is well taken and we must emphasize that this is more of a comparative tool (species/ areas).

Another minor thing I took note of was that the duration of the closed (measurement) and flushing periods of the intermittent-flow respirometry technique was apparently always kept the same (5 min flushing, 10 min closed; detailed in table S1) across the wide range of experimental test temperatures (5 to 22’C). As the animals would have respired faster at the higher temperatures (as the presented data also shows), oxygen levels inside the respirometry chambers would have dropped (much) more at the higher vs. lower temperatures. I would have recommended that at least close durations were adjusted based on the trial temperature, so the drop in oxygen remained similar at the various temperatures. The authors detail in Table S1 that oxygen levels inside the respirometry chambers never fell below 85% air saturation at a given temperature, which is still high, so presumably the fixed closed duration has not affected estimates of SMR, as 85% air saturation is above Pcrit for both species, although red abalone does show a strong sensitivity to hypoxia at 50% air saturation in terms of the optimal temperature of the metabolic index (Fig. 5B).

We set the measuring time of 5 minutes to get decent drawdown data for the colder temperatures (5/7/10 degrees C). As we tested warmer temperatures the drawdown data never exceeded 85% saturation, so we didn’t adjust the length. We did however run two trials with a ten-minute measurement period for 7 deg C runs. We mentioned that on line 517 where we define the measurement period as 5/10 and flush period as ten minutes). We have updated the supplementary table S1 to reflect this.

Overall – without having seen the raw respirometry data – the experiments themselves

appear to be well conducted, although the ecological relevance of the produced data and their use in predicting habitat suitability are questionable, given the acute nature of the lab thermal exposures vs. the presumably more stable (acclimated) thermal environment in nature.

Raw respirometry data attached to this response to reviews submission
